# Modulation of endothelial-to-mesenchymal transition via NRP-1 targeting with melittin attenuates pulmonary fibrosis

**DOI:** 10.1016/j.mtbio.2025.102659

**Published:** 2025-12-09

**Authors:** Ming Hu, Yingying Wan, Jiakang Chen, Chengwei Zhang, Shuze Li, Bingbing Shan, Ling Wu, Xiang Yu

**Affiliations:** a State Key Laboratory of Digital Medical Engineering, School of Biomedical Engineering, Hainan University, China; b Key Laboratory of Biomedical Engineering of Hainan Province, One Health Institute, Hainan University, China; c Hubei Key Laboratory of Tumor Microenvironment and Immunotherapy, China Three Gorges University, China; d Sanya Yazhou Bay Science and Technology City Hospital, Sanya, China

**Keywords:** Melittin, NRP-1, EndMT, Peptide-lipid nanoparticles

## Abstract

The transforming growth factor-β (TGF-β) signaling pathway is a central driver in the pathogenesis of pulmonary fibrosis (PF), and strategies targeting this pathway demonstrate therapeutic potential. However, the ubiquitous blockade of TGF-β signaling is associated with detrimental effects due to its pleiotropic involvement in physiological processes. Here, we demonstrate that blocking neuropilin-1 (NRP-1), a high-affinity TGF-β co-receptor, with host defense peptide melittin (MLT) attenuates PF progression. Molecular docking and surface plasmon resonance (SPR) demonstrated direct binding between MLT and NRP-1. *In vitro* studies revealed that MLT selectively targeted endothelial cells with high NRP-1 receptor expression, suppressing endothelial-to-mesenchymal transition (EndMT) via inhibition of TGF-β/Smad and MAPK pathways activation. In a bleomycin-induced PF model, elevated NRP-1 expression enhanced fluorescence-labeled MLT targeting in fibrotic lungs, enabling MLT to exert dose-dependent anti-fibrotic effects through EndMT suppression and significantly improve survival in PF mice. Furthermore, *in vivo* imaging showed that MLT-loaded peptide-lipid nanoparticles (M-pLNPs) formed a depot sustaining lung fluorescence for over 24 h, starkly contrasting with free MLT clearance within 6 h, which may contribute to comparable anti-fibrotic efficacy at a lower dose. Therefore, our results suggest a novel mechanism involving co-receptor blockade for the anti-fibrotic effect of MLT, highlighting its potential as a therapeutic candidate for PF.

## Highlights

• MLT selectively targets endothelial cells undergoing EndMT via NRP-1 receptor.
• MLT significantly inhibited EndMT through dual inhibition of TGF-β/Smad and MAPK pathways.
• MLT attenuates BLM-induced PF in a dose-dependent manner.
• Nanoparticle-based delivery of MLT potentiates anti-PF efficacy at a reduced dose.

## Graphical abstract

**Figure:**
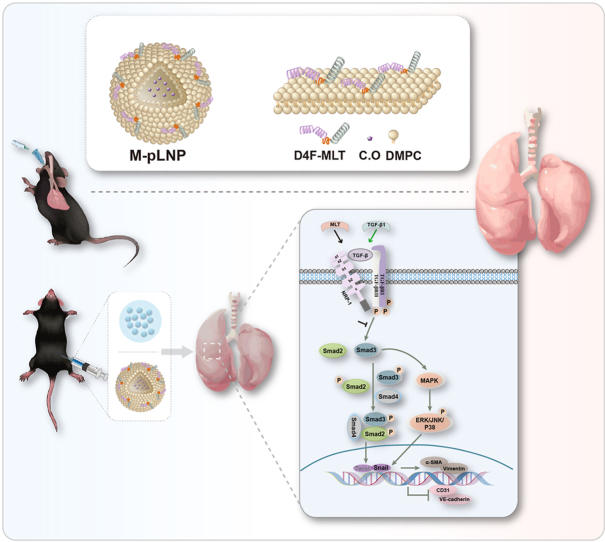

## 1 Introduction

Pulmonary fibrosis (PF) is a chronic progressive lung disease that mainly occurs in elderly individuals, with a median survival ranging from 3 to 5 years. Moreover, the prevalence of PF is increasing rapidly due to post-COVID-19 pulmonary abnormalities. PF is characterized by the formation of many fibroblast foci, which leads to considerable extracellular matrix accumulation and remodeling of the lung interstitium [1,2]. Endothelial-to-mesenchymal transition (EndMT), a well-established source of fibroblasts in PF, contributes to fibrosis development by enabling lung endothelial cells to transform into a significant number of fibroblasts [3,4]. During EndMT, driven by factors like TGF-β, endothelial cells gradually lose the expression of specific endothelial markers, such as CD31 and VE-cadherin, and acquire mesenchymal cell products, such as vimentin and alpha-smooth muscle actin (α-SMA) [5]. Accordingly, TGF-β inhibition has motivated significant therapeutic interest for fibrotic diseases, given its central role in regulating this process [6].

Although recent studies have shown that inhibition of TGF-β activation and signaling clearly leads to significant improvements in related diseases in mouse models, the clinical effect is not as expected [7]. This may be due to the extensive biological activity of TGF-β and its involvement in many normal physiological processes, as non-selective blockade of TGF-β may have adverse consequences [8,9]. Notably, in addition to the canonical ligand-receptor combinations, cell surface co-receptors also play a critical role in TGF-β signaling. By interacting with classical TGF-β receptors, co-receptors can influence the recruitment of complex components, protein stability, and intracellular trafficking dynamics [10]. Since these modulators drive excessive pathological signaling, targeting TGF-β co-receptors is a promising strategy to employ context-specific inhibition, thereby mitigating toxicity while preserving physiological TGF-β functions [11]. This approach contrasts with the systemic adverse effects frequently observed with direct TGF-β or receptor blockade, as supported by preclinical and mechanistic studies [12]. Neuropilin-1 (NRP-1) is a multifunctional, transmembrane, non-tyrosine kinase surface glycoprotein originally discovered by Fujisawa and colleagues as an axon adhesion protein in the developing frog nervous system [13]. Numerous studies have shown that NRP-1 not only serves as a high-affinity receptor for both the latent and active forms of TGF-β1 but also activates Smad signal transduction in response to TGF-β stimulation [[14], [15], [16], [17]]. In addition, NRP-1 may also regulate TGF-β non-Smad signaling, which further induces the deterioration of PF [18]. Therefore, targeting NRP-1 to modulate TGF-β and its downstream signaling pathways is a promising strategy for the treatment of PF.

Melittin (MLT) is a natural cationic host defense peptide that is the main bioactive component in the venom of honeybees (*Apis Mellifera*) and has 26 amino acid residues (GIGAVLKVLTTGLPALISWIKRKRQQ). It consists of a hydrophobic N-terminal region (1–20 amino acids) and a hydrophilic C-terminal region (21–26 amino acids) containing several positively charged amino acid residues [19,20]. Many studies have shown that this natural compound has pharmacological effects on a variety of diseases, such as cancer, inflammation, and bacterial infections [[21], [22], [23]]. It has been reported that MLT inhibits epithelial-mesenchymal transition and tumor invasion in various cancer cells [[24], [25], [26]]. Only recently has the anti-fibrotic efficacy of MLT begun to receive attention [27]. However, the role and mechanism of MLT in PF remain unclear. Moreover, its rapid systemic clearance restricts lung exposure and overall efficacy. It has also been reported that peptides containing R/K/XXR/K sequences can target NRP-1 [28,29]. Therefore, we speculate that MLT may have the potential to target NRP-1 and regulate the TGF-β signaling pathway to play an anti-fibrotic role. In the present study, we used a combination of *in vitro* and *in vivo* approaches to determine the anti-fibrotic effects and mechanism of MLT in PF. To modulate the pharmacokinetics of MLT for extended duration of action in the lungs, we engineered MLT-loaded peptide-lipid nanoparticles (M-pLNPs) and evaluated their therapeutic performance. Notably, this strategy significantly reduced the required dosage without compromising efficacy, demonstrating the practical advantage of M-pLNPs for clinical application.

## 2 Materials and methods

### 2.1 Materials

Peptides and proteins: MLT (GIGAVLKVLTTGLPALISWIKRKRQQ-NH_2_), D4F-peptide (DWFKAFYDKVAEKFKEAF-NH_2_), D4F-MLT (DWFKAFYDKVAEKFKEAFGSGGIGAVLKVLTTGLPALISWIKRKRQQ-NH2), and Rhodamine B-labeled MLT and D4F-MLT were synthesized by Apeptide Co., Ltd. (Shanghai, China). NRP-1 protein was purchased from MCE (New Jersey, USA). TGF-β1 was obtained from Pepro Tech (New Jersey, USA). Cell culture and biochemical reagents: Fetal bovine serum (FBS), penicillin/streptomycin and Dulbecco's modified Eagle's medium (DMEM) were purchased from Gibco (Massachusetts, USA). Endothelial cell growth supplement (ECGS) was purchased from ScienCell (California, USA). Dimethyl sulfoxide (DMSO) was purchased from Sigma-Aldrich (Michigan, USA). Molecular biology reagents: Total RNA Extraction Reagent, HiScript® III RT SuperMix for qPCR (+gDNA wiper), and Taq Pro Universal SYBR qPCR Master Mix were purchased from Vazyme Biotech Co., Ltd (Nanjing, China). Antibodies: VE-cadherin, β-actin and HRP-labeled antibodies were purchased from Boster Company. CD31, α-SMA, vimentin, Snail, Twist, Smad4, JNK, p-JNK, ERK and p-ERK were purchased from Proteintech (Wuhan, Hubei, China). Smad2/3 and p-Smad2/3 were purchased from Affinity Biosciences Inc (Changzhou, China). NRP-1, Goat Anti-Rabbit IgG H&L (Alexa Fluor® 594), Goat Anti-Rat IgG H&L (Alexa Fluor® 647), α-SMA and COL-I antibodies were purchased from Abcam. Other materials: Bleomycin Sulfate was obtained from Macklin Biochemical Co., Ltd. (Shanghai, China).

### 2.2 Animals and cells

Specific pathogen-free (SPF) C57BL/6 mice (6–8 weeks old) were purchased from the Yancheng Biotechnology Co., Ltd. (Guangzhou, Guangdong, China). All animal experiments were approved by the Animal Care and Use Committee of Hainan University. Human umbilical vein endothelial cells (HUVECs) were purchased from by PriCells Biomedical Technology Co., Ltd (Wuhan, China). Cells were cultured in Dulbecco's modified Eagle medium (DMEM) supplemented with 10 % FBS, 1 % penicillin/streptomycin, and 1 % ECGS at 37 °C in a humidified atmosphere containing 5 % CO_2_.

### 2.3 Molecular docking

The three-dimensional (3D) structures of MLT (PDB ID:2MLT) and NRP-1 (PDB ID:6TKK) were both downloaded from the RCSB Protein Data Bank (https://www.rcsb.org/). Molecular docking was used to investigate the binding mode between MLT and the NRP-1 protein with the ZDOCK server (https://zdock.umassmed.edu/), and the most stable complex was selected from the top 10 complexes obtained. Protein-peptide interactions within the complex were visualized and analyzed using the PyMOL molecular graphics system (https://pymol.org).

### 2.4 SPR analysis

To measure the interaction between the stationary protein (NRP-1) and the mobile peptide (MLT), monitoring was performed using a biomolecular interaction analyzer (Biacore 8K, Cytiva, USA).The activator was prepared by mixing 400 mM EDC and 100 mM NHS immediately prior to injection. The CM5 sensor chip was activated for 420 s with the mixture at a flow rate of 10 μL/min. Dilute NRP-1 protein to 20 μg/mL in immobilization buffer, then injected to sample channel Fc2 at a flow rate of 10 μL/min, and typically result in immobilization levels of 12600 RU, the reference channel Fc1 does not need ligend immobilization step. The chip was deactivated by 1 M Ethanolamine hydrochloride at a flow rate of 10 μL/min for 420 s. Dilute MLT with the same analyse buffer to 8 conxentrations (50, 25, 12.5, 6.25, 3.13, 1.56, 0.78 and 0.1 μM). MLT was injected Fc1-Fc2 at a flow rate of 20 μL/min for an association phase of 100 s, followed by 180 s dissociation. The association and dissociation process were all handling in the analyse buffer. Repeat 8 cycle of analyte according concentrations in ascending order.

### 2.5 Cell viability assay

Cell viability was assessed using the MTS assay (Promega). Briefly, HUVECs were seeded at 5000 cells/well in triplicate in a 96-well plate. When the density of the cells reached approximately 80 %, MLT at different concentrations was added, and the cells were incubated for 24 h. Then, 20 μL of MTS reagent was added to each well, followed by incubated for 2 h. Absorbance was measured at 490 nm using a microplate reader.

### 2.6 In vitro cell targeting

To assess the NRP-1 receptor-targeting ability of MLT, HUVECs were seeded into 96-well plates at a density of 1.5 × 10^4^ cells per well and cultured overnight in complete growth medium. TGF-β1 (10 ng/mL) was added to all groups except the control group and incubated for 48 h. Subsequently, each group was treated with the RPARPAR peptide (0.5 mM) for 1 h, followed by the addition of MLT-RhB (2 μg/mL) for an additional hour. For flow cytometry, cells were processed and analyzed using a flow cytometer (Dakewe Biotech Co., Ltd., China), and data were analyzed with FlowJo software (FlowJo, Ashland, OR, USA). For immunofluorescence, following the same treatment protocol, cells were fixed with 4 % paraformaldehyde and immunostained. NRP-1 was detected using an anti-NRP-1 primary antibody and an Alexa Fluor® 647-conjugated secondary antibody. Nuclei were stained with DAPI. Imaging was performed using a SpinSR10 spinning disk confocal super-resolution microscope (Olympus, Japan) with the following excitation wavelengths: 405 nm for DAPI; 535 nm for RhB; and 640 nm for NRP-1.

### 2.7 Western blot

Equal amounts of proteins from cell lysates were separated by 12 % SDS‒PAGE and transferred to PVDF membranes. The membrane was blocked with nonfat dry milk and probed with primary antibodies against NRP-1 (1:1000), β-actin (1:6000), CD31 (1:6000), α-SMA (1:5000), vimentin (1:6000), VE-cadherin (1:2000), Smad2/3 (1:2000), p-Smad2/3 (1:2000), Smad4 (1:4000), JNK (1:6000), p-JNK (1:2000), ERK (1:1000), and p-ERK (1:5000). The membrane was then incubated with secondary antibodies and washed. Proteins were detected using enhanced chemiluminescence (ECL) reagent (Kerui Institute of Biotechnology, Wuhan, China) and a ChemiScope 6100 chemiluminescence imaging system (Clinx, Shanghai, China). Band intensity was quantified using ImageJ.

### 2.8 Immunofluorescence staining

HUVECs were washed three times with PBS and then fixed with 4 % paraformaldehyde at room temperature. The cells were then blocked in 1 % BSA diluted in PBS for 30 min and incubated with 0.2 % Triton-X 100 for 5 min at 4 °C. After that, the cells were incubated overnight at 4 °C with anti-CD31 antibody (1:200), anti-VE-cadherin antibody (1:100), anti-α-SMA antibody (1:200), anti-Vimentin antibody (1:200), anti-Twist antibody (1:150), and anti-Snail antibody (1:150). After three washes, cells were incubated with Alexa Fluor® 594-conjugated goat anti-rabbit antibody (1:400) or Alexa Fluor® 647-conjugated goat anti-mouse antibody (1:400) for 1 h at room temperature. The nuclei were stained with DAPI.

For immunofluorescence staining of the lung, the upper lobe of the left lung was excised, fixed with 4 % paraformaldehyde, dehydrated with 30 % sucrose, and embedded in an OCT compound. Tissue sections, 10 μm in thickness, were obtained using a cryostat (Dakewe Biotech Co., Ltd., China) and subsequently mounted on poly L-lysine-coated slides. All specimens were subjected to an overnight incubation at 4 °C with primary antibodies against CD31 (1:200), VE-cadherin (1:100), α-SMA (1:200), COL-I (1:200), vimentin (1:200), Twist (1:150), and Snail (1:150). Following three rounds of washing, the samples were then incubated for 1 h at room temperature with Alexa Fluor® 594-conjugated goat anti-rabbit secondary antibody (1:400) or Alexa Fluor® 647-conjugated goat anti-mouse secondary antibody (1:400). DAPI was used to stain the cell nuclei. Images were captured by the SpinSR10 spinning disk confocal super resolution microscope (Olympus, Japan).

### 2.9 RT-qPCR

HUVEC were seeded at a concentration of 3 × 10^5^ cells/mL into 6-well plates and allocated into four groups: a control group, a TGF-β1 group, a low-dose MLT group (0.5 μg/mL), and a high-dose MLT group (2 μg/mL). Following an overnight incubation, the cells were treated with TGF-β1 (10 ng/mL) and subsequently exposed to either low-dose or high-dose MLT for 48 h. Total RNA was extracted from HUVECs using TRIzol reagent and reverse transcribed to cDNA using a reverse transcription kit. We performed RT-qPCR reactions with SYBR Green reaction mixture (Vazyme, China) in a StepOnePlus RT-qPCR system (Applied Biosystems, Foster City, CA). Relative gene expression was calculated using β-actin as an internal control. Primer sequences are listed in Table S1.

### 2.10 In vitro scratch assay

The cells were inoculated into 6-well plates and allowed to grow until they reached approximately 100 % confluence, at which point they were deemed ready for experimental procedures. To initiate the scratch assay, a clean, sterile 200 μL pipette tip was used to create a single, linear wound across the surface of the cell monolayer in each well. Following the creation of the scratch, the cell cultures were gently washed with phosphate-buffered saline (PBS) to remove any debris or detached cells. Subsequently, the cells were incubated in fresh culture medium that supplemented with or without TGF-β1 and MLT. The progression of cell migration into the wounded area was monitored and documented at various intervals (0, 12, and 24 h) using the BioTek Cytation 5 multimodal imaging system (BioTek, Winooski, VT, USA).

### 2.11 Cell migration assay

Migration assays were performed in triplicate using Transwell chambers for 24-well plates (0.8 μm pore size, LABSELECT). HUVECs (5 × 10^4^) were plated in 200 μL of DMEM medium supplemented with 0.1 % FBS in the upper chamber. The lower chamber was filled with 700 μL of DMEM medium with supplemented 30 % FBS. After culturing for 12 or 24 h, the noninvaded cells were mechanically removed with a cotton swab. The invaded cells on the underside of the membrane were fixed with 4 % formalin and stained with 0.1 % crystal violet for visualization. Cells were counted in ten respective microscopic fields (40 magnification) and photographed.

### 2.12 Synthesis and characterization of M-pLNPs

A mixture of DMPC (3 μmol) and C.O (0.2 μmol) in chloroform (300 μL, AR grade) was dried under nitrogen to form a uniform lipid film. Then, 1 ml PBS was added to the dried film, and the mixture was vortexed for at least 5 min to redissolve the adsorbed product. Subsequently, the mixture was sonicated for about 1 h at 48 °C until the solution is as clear as possible. D4F-MLT fusion peptide was dissolved in 1 ml double distilled water. This peptide was added to the lipid emulsion, and then stored overnight at 4 °C. After being concentrated by centrifugal filter units (30 Kd, Millipore, USA), the nanoparticles were purified using a fast protein liquid chromatography system with a HiLoad 16/60 Superdex 200 pg column (General Electric Healthcare, NY, USA). The particle size and zata potential of M-pLNPs were measured using Zetasizer Nano ZS (Malvern Instruments). The morphology of M-pLNPs was examined using transmission electron microscopy (TECNAI G2, FEI Company, OR, USA). The ultraviolet–visible absorption spectra of M-pLNP-Rhb and M-pLNP were recorded using a UV–Vis spectrophotometer (Shimadzu Instrument Co., LTD, Suzhou, China). The fluorescence spectra were measured with an F-7000 fluorescence spectrophotometer (Hitachi, Ltd., Tokyo, Japan).

### 2.13 Animal treatment experiments

To evaluate the anti-fibrotic efficacy of MLT, two sequential therapeutic experiments were performed in BLM-induced PF mouse model. In the first experiment, male C57BL/6 mice (n = 20) were randomized into four groups: normal control group, BLM group, low-dose group (MLT-L, 0.5 mg/kg), high-dose MLT treatment group (MLT-H, 2 mg/kg). Following intratracheal instillation of BLM (5 mg/kg) or saline on day 0, MLT were administered daily via intraperitoneal injection from day 7. In the second experiment (n = 30), mice were divided into six groups: normal control, BLM model, pLNPs alone, low-dose free MLT (MLT-L, 0.5 mg/kg), high-dose free MLT (MLT-H, 2 mg/kg), and M-pLNPs (0.5 mg/kg as MLT). After identical PF induction, treatments (free MLT or M-pLNP) were administered every other day from day 7. All mice were euthanized on day 21 for collection of serum and lung tissue.

### 2.14 Ex vivo fluorescence imaging

To evaluate the biodistribution and lung targeting of MLT and M-pLNP, two fluorescence imaging experiments were performed in a BLM-induced PF model. In the first study, normal and PF mice (n = 6) received a single intraperitoneal injection of MLT-RhB (or PBS control), and were euthanized 30 min later for collection of major organs. In the second study, PF mice were randomly divided into two groups receiving either MLT-RhB or M-pLNP-RhB. Comprehensive *in vivo* whole-body fluorescence imaging was performed at multiple time points (10 min, 30 min, 1 h, 3 h and 6 h) after administration. Additionally, *ex vivo* analysis was conducted by collecting blood samples and lung tissues at four critical time points (10 min, 30 min, 1 h, 6 h, 12 h, and 24 h). All imaging was performed using an IVIS Lumina XRMS system, *in vivo* and *ex vivo* samples were imaged through RhB channels (Ex/Em = 545/570 nm). Mean fluorescence intensity was used to quantitatively assess tissue distribution and lung accumulation.

### 2.15 Histological analysis

On day 21 post-treatment, lung tissues were collected, fixed in 4 % paraformaldehyde, dehydrated, and paraffin-embedded. Consecutive sections were then prepared for hematoxylin and eosin (H&E) and Masson's trichrome staining. Imaging was performed using a PanoBrain microscope (Laishang Meca, Guangzhou, China). PF was semi-quantitatively assessed on five H&E-stained sections spaced 1 mm apart, employing the Ashcroft scoring system (range: 0, normal; 8, total fibrosis). The final fibrosis score for each sample was expressed as the mean of all evaluated fields.

### 2.16 Hydroxyproline assay

The hydroxyproline (HYP) content in lung tissues was quantified using a commercial assay kit (Jiancheng, Nanjing, China). Briefly, tissue samples were homogenized in trichloroacetic acid. The resulting homogenate was washed with distilled water and then hydrolyzed in hydrochloric acid. After neutralizing the hydrolysate with NaOH, chloramine-T and dimethylaminobenzaldehyde were sequentially added. The absorbance at 550 nm was measured, and the hydroxyproline content was expressed as micrograms per milligram of wet lung tissue (μg/mg).

### 2.17 Micro-CT

The mice were anesthetized by respiration with isoflurane (R510-22-10; RWD, Shenzhen, China). To assess lung damage in mice in a clinically relevant manner, the mice were placed in a prone position, chest CT images were acquired 21 days after establishment of a mouse model of PF using a Quantum GX2 Micro-CT Imaging System (PerkinElmer). The Micro-CT utilized an X-ray filter (0.06 mm Cu + 0.5 mm Al) at 90 KV, 0.088 mA.

### 2.18 Pulmonary function test

Pulmonary function tests in experimental animals were evaluated by an invasive Animal Pulmonary Function Testing and Analysis system (flexiVent, SCIREQ, Canada). Anesthetized mice were dissected into the neck skin and intubated using a 22 G indwelling needle. Pulmonary function parameters were recorded to assess pulmonary function.

### 2.19 Statistical analysis

All experiments were performed in at least three independent replicates. Data are reported as mean ± SEM. Statistical significance between two groups was evaluated using an unpaired Student's t-test. For comparisons involving multiple groups, one-way analysis of variance (ANOVA) was employed. The comparison of survival rates between groups was plotted using the Kaplan-Meier method to create survival curves, and statistical analysis was performed using the Log-rank test. In all cases, *p* < 0.05 was considered statistically significant. GraphPad Prism software (version 8.0.2) was used for all statistical analyses.

## 3 Results

### 3.1 MLT targets EndMT cells via NRP-1 receptor

**Fig. 1:**
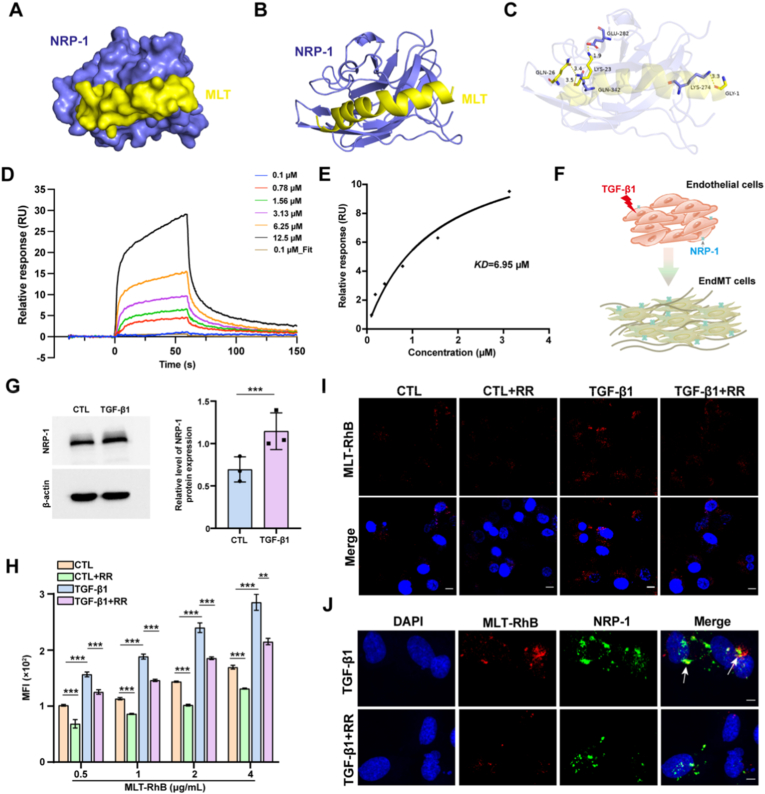
NRP-1-dependent targeting of MLT to EndMT cells. (A) Surface representation of the docking conformation between MLT (yellow) and NRP-1 (violet). (B) Cartoon representation of the MLT (yellow) and NRP-1 (violet) complex. (C) The interacting residues of MLT (yellow) and the NRP-1 (violet) complex are shown in sticks representation. The hydrogen bonds are shown as yellow dotted lines. (D) Representative SPR sensorgrams for the binding interaction of MLT to immobilized NRP-1. (E) Steady state affinity fitting of MLT binding to NRP-1. (F) Schematic of the *in vitro* model for inducing EndMT. (G) Quantitative analysis of NRP-1 protein expression in HUVECs. The original western blot image is provided in Fig. S2. (H) Quantitative analysis of mean fluorescence intensity (MFI) of RhB in HUVECs treated with the indicated concentrations of MLT-RhB. (I) Confocal microscopy images showing the cellular uptake of MLT-RhB in HUVECs. Scale bar: 10 μm. (J) Co-localization of MLT with NRP-1 receptor in HUVECs visualized by confocal imaging. White arrows indicate representative co-localization sites. Scale bar: 5 μm; Blue: DAPI; Red: RhB; Green: NRP-1. Data are presented as the mean ± SEM (n = 3); ∗∗*p* < 0.01, and ∗∗∗*p* < 0.001.

Based on the established high affinity of R/K/XXR/K motifs for the NRP-1 receptor [28], we employed molecular docking to validate the binding capability of MLT to NRP-1. The docking conformations of NRP-1 (PDB ID:6TKK; in violet representation) and MLT (PDB ID: 2MLT, in yellow representation) are illustrated on the surface (Fig. 1A) and as a cartoon (Fig. 1B). As shown in Fig. 1C, a zoomed view represented the MLT binding mode into the b1 domain of NRP-1, highlighting interactions with the key amino-acid residues. This analysis revealed that hydrogen bond interactions were observed between GLY-1 of MLT and LYS-274 (bond length: 3.3 Å) of NRP-1, LYS-23 of MLT and GLN-342 (bond length: 3.5 Å) of NRP-1, LYS-23 of MLT and GLU-282 (bond length: 1.9 Å) of NRP-1, and GLN-26 of MLT and GLN-342 (bond length: 3.4 Å) of NRP-1. To further verify the interaction between MLT and NRP-1, the binding affinity between MLT and NRP-1 receptor was quantitatively analyzed by surface plasmon resonance (SPR). After immobilization on the CM5 chip, NRP-1 protein was allowed to bind to different concentrations of MLT peptide, and the binding kinetics was measured in real time. The affinity constant (KD) was 6.95 × 10^−6^ M, which confirmed that MLT could effectively target and bind to NRP-1 receptor (Fig. 1D and E). On the basis that MLT can specifically target NRP-1 receptor, we further hypothesized that MLT may specifically target cells undergoing EndMT by binding to the highly expressed NRP-1 receptor on the surface of endothelial cells. Before assessing the functional consequences of this targeting, it was essential to determine potential cytotoxicity. Therefore, we measured HUVEC viability using the MTS assay following MLT treatment. The results suggested that at concentrations ranging from 0.125 to 4 μg/mL, MLT was not cytotoxic to HUVECs, whereas 8 μg/mL MLT started to induce cellular toxicity (Fig. S1). Therefore, the 0.125–4 μg/mL MLT concentration range was selected for subsequent experiments.

Given that the expression of NRP-1 is positively correlated with the degree of EndMT [30], an *in vitro* model of EndMT was established by treating HUVECs with TGF-β (Fig. 1F). Subsequently, we performed western blot analysis to detect the protein expression of NRP-1 in cells during EndMT. The results showed that the expression of the NRP-1 protein was significantly upregulated after TGF-β1-induced EndMT in HUVECs (Fig. 1G). Therefore, we utilized TGF-β1-induced EndMT cell model to further verify the targeting effect of MLT on NRP-1. Specifically, HUVECs were pretreated with an NRP-1-targeting peptide (RPARPAR, RR) to block the NRP-1 receptor in advance and then incubated with Rhodamine B-labeled MLT (MLT-RhB). As shown in Fig. 1H, flow cytometry (FCM) data revealed that the mean fluorescence intensity (MFI) of RhB in the TGF-β1 group was significantly higher than that in the control group (CTL), indicating that the elevated NRP-1 receptor levels can enhance the uptake of MLT-RhB. In addition, after RR peptide pretreatment, the MFI of RhB in both the TGF-β1 and CTL groups was significantly reduced. Confocal imaging also showed that the uptake capacity of MLT-RhB in the TGF-β1 group was greater than that in the CTL group, and pretreatment with NRP-1-targeting RR peptide significantly attenuated the uptake of MLT-RhB (Fig. 1I). Furthermore, direct colocalization of MLT with NRP-1 receptors was observed, providing direct visual evidence of the spatial association between MLT and the NRP-1 receptor on endothelial cells undergoing EndMT (Fig. 1J). Taken together, these results demonstrate that MLT can effectively target endothelial cells undergoing EndMT via the NRP-1 receptor.

### 3.2 MLT inhibits TGF-β1-induced EndMT *in vitro*

**Fig. 2:**
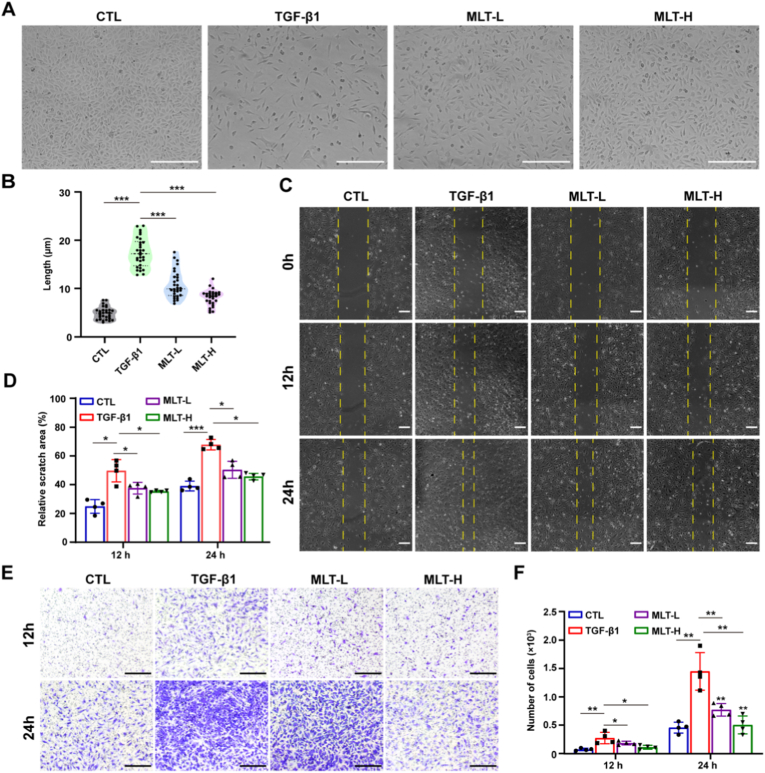
MLT attenuates TGF-β1-induced morphological and migratory changes in HUVECs. (A) Morphological changes in HUVECs under different conditions. Scale bar: 100 μm. (B) Quantitative analysis of cell length of 30 randomly selected HUVECs. (C) Wound healing assay, Scale bar: 200 μm. (D) Quantitative analysis of wound closure. (E) Transwell assays were used to detect the migration of HUVECs, Scale bar: 200 μm. (F) Quantitative analysis of cell migration in the transwell assay. Data are presented as the mean ± SEM (n = 4); ∗*p* < 0.05, ∗∗*p* < 0.01, and ∗∗∗*p* < 0.001.

**Fig. 3:**
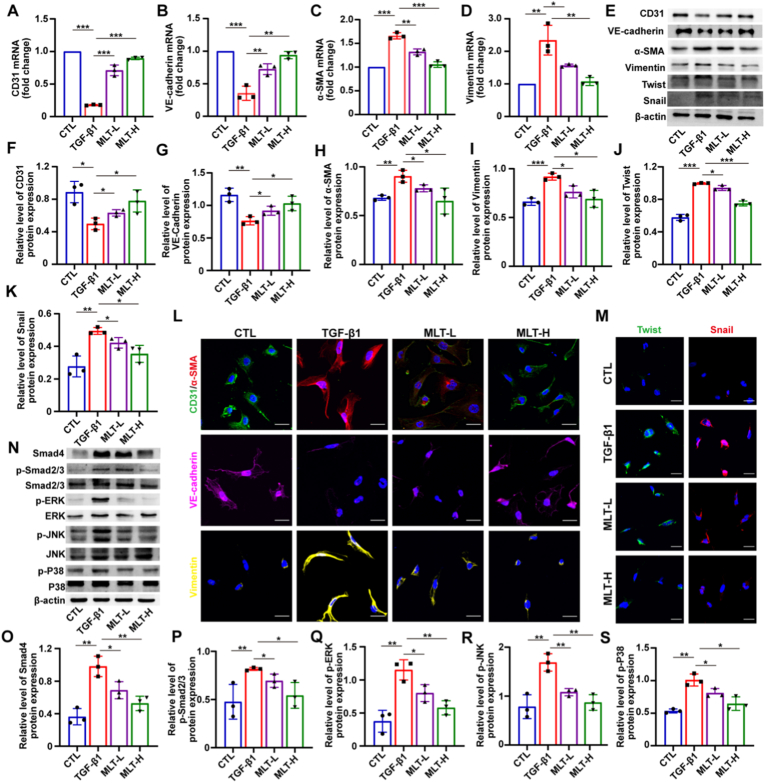
MLT attenuates TGF-β1-induced EndMT by inhibiting the TGF-β/Smad and MAPK pathways in HUVECs. (A–D) The mRNA levels of endothelial (CD31, VE-cadherin) and mesenchymal (α-SMA, vimentin) markers were determined by RT-qPCR. (E) EndMT-related protein expression of CD31, VE-cadherin, α-SMA, vimentin, Twist, and Snail was examined by western blot. The original western blot images are provided in Fig. S3 (F–K) Quantitative analysis of CD31, VE-cadherin, α-SMA, vimentin, Twist, and Snail protein levels (relative to β-actin). (L) Immunofluorescence staining of EndMT markers in HUVECs. Scale bar: 50 μm; Blue: DAPI; Green: CD31; Magenta: VE-cadherin; Red: α-SMA; Orange: vimentin. (M) Nuclear transcription factor immunofluorescence during EndMT in HUVECs. Scale bar: 50 μm; Blue: DAPI; Green: Twist; Red: Snail. (N) Protein levels of key components in the TGF-β/Smad and MAPK pathways were assessed by western blot. The original western blot images are provided in Fig. S4 (O–S) Quantitative analysis of Smad4/β-actin, p-Smad2/3/Smad2/3, p-ERK/ERK, p-JNK/JNK and p-P38/P38 protein levels. Data are presented as the mean ± SEM (n = 3); ∗*p* < 0.05, ∗∗*p* < 0.01, and ∗∗∗*p* < 0.001.

To further investigate the role of MLT in EndMT, we induced EndMT *in vitro* by stimulating HUVECs with TGF-β1 (10 ng/mL) for 48 h. As shown in Fig. 2A, TGF-β1 stimulation for 48 h changed the morphology of HUVECs from a characteristic cobblestone shape to an elongated and spindle-like phenotype. Treatment with MLT partially reversed the TGF-β1-induced morphological changes (Fig. 2A and B). Next, we performed cell scratch migration and transwell migration assays to show that TGF-β1 stimulated cell migration and motility in HUVECs, while MLT treatments significantly inhibited cell migration and motility (Fig. 2C–F). Furthermore, the inhibitory effect appeared most pronounced at the highest MLT dose, though statistical comparison between doses was not performed. RT-qPCR analysis of TGF-β-treated HUVECs revealed increased expression of the fibroblast markers α-SMA and vimentin, alongside decreased expression of the EC markers CD31 and VE-cadherin, indicating that the cells underwent EndMT. However, MLT treatment dose-dependently reversed these changes, decreasing α-SMA and vimentin mRNA levels while increasing CD31 and VE-cadherin mRNA levels (Fig. 3A–D). To further confirm that MLT inhibits TGF-β-induced EndMT, we used western blot analysis to detect protein levels of endothelial and mesenchymal markers. This analysis revealed that MLT treatment significantly upregulated endothelial marker proteins (CD31 and VE-cadherin), while downregulating mesenchymal markers (α-SMA, vimentin) and nuclear transcription factors (Twist, Snail) (Fig. 3E–K). Immunofluorescence staining corroborated these findings, demonstrating that MLT attenuated TGF-β-mediated EndMT. Specifically, MLT reversed the loss of CD31 and VE-cadherin, downregulated α-SMA and vimentin expression, and reduced Twist and Snail nuclear expression (Fig. 3L and M). Collectively, these experiments demonstrate that MLT dose-dependently inhibits TGF-β-induced EndMT.

Given the established involvement of Smad and downstream MAPK signaling in TGF-β-induced EndMT, we sought to determine whether MLT exerts its inhibitory effect on EndMT through targeted inhibition of the TGF-β/Smad and MAPK signaling pathways. Western blot results showed that TGF-β1 increased the expression levels of Smad4 and p-Smad2/3 in HUVECs, whereas MLT treatment reversed these increases (Fig. 3N–P). Furthermore, we assessed the activation status of downstream MAPK pathways. The results showed that TGF-β1 activated all three MAPK pathways, including ERK, p38, and JNK, while MLT suppressed their activation (Fig. 3Q–S). Taken together, these results indicated that MLT markedly inhibited TGF-β1-induced EndMT by suppressing the activation of TGF-β/Smad and MAPK signaling.

### 3.3 MLT accumulates in fibrotic lungs by targeting NRP-1

**Fig. 4:**
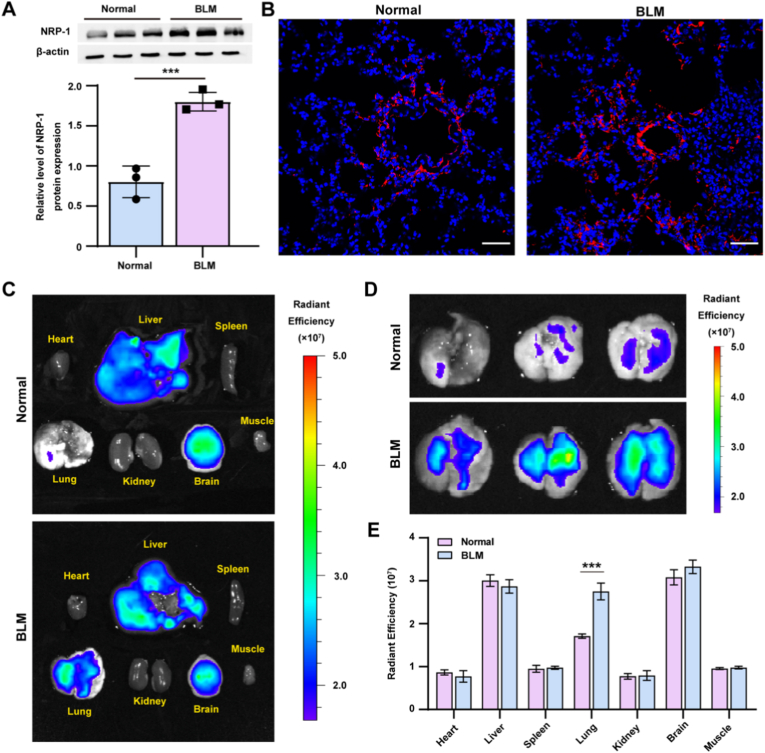
*In vivo* biodistribution of MLT in mice. (A) NRP-1 protein expression in lung tissues was assessed by western blot. The original western blot image is provided in Fig. S5. (B) Representative images of immunofluorescence staining of lung tissue. Scale bar: 100 μm; Blue: DAPI; Red: NRP-1. (C) *Ex vivo* fluorescence imaging of major organs harvested 30 min post-injection. (D) Fluorescence images of MLT-RhB in lung tissues. (E) MFI analysis of MLT-RhB in lung tissues. Data are presented as the mean ± SEM (n = 3); ∗∗∗*p* < 0.001.

Several studies have shown that the expression of NRP-1 is upregulated in fibrotic organs, which is consistent with the degree of fibrosis in these organs [18,31]. To evaluate the role of NRP-1 expression in the progression of PF, BLM-induced PF models were constructed and used for the subsequent experiments. First, we assessed NRP-1 protein expression in fibrotic versus normal lung tissues by western blot and immunofluorescence. Both methods consistently revealed markedly higher NRP-1 protein expression in the lung tissue of PF mice than in that of normal mice (Fig. 4A and B). To evaluate the biodistribution and targeting capability of MLT, MLT-RhB was intraperitoneally administered, and the fluorescence intensity in organs was assessed using a small animal *in vivo* imaging system. Strikingly, PF mice exhibited significantly stronger pulmonary fluorescence signals within 30 min post-injection compared to healthy mice (Fig. 4C and D), indicating preferential accumulation of MLT in fibrotic lungs. Quantitative analysis confirmed this observation, with PF lungs showing ∼1.5-fold higher radiant efficiency than control (Fig. 4E). A low fluorescence signal was also detected in the lungs of normal mice, consistent with the basal expression of NRP-1 on pulmonary endothelial cells. These collective findings demonstrate that MLT preferentially accumulates in fibrotic lung tissue through active targeting of the upregulated NRP-1 receptor.

### 3.4 Therapeutic suppression of PF by MLT in BLM-induced mice

**Fig. 5:**
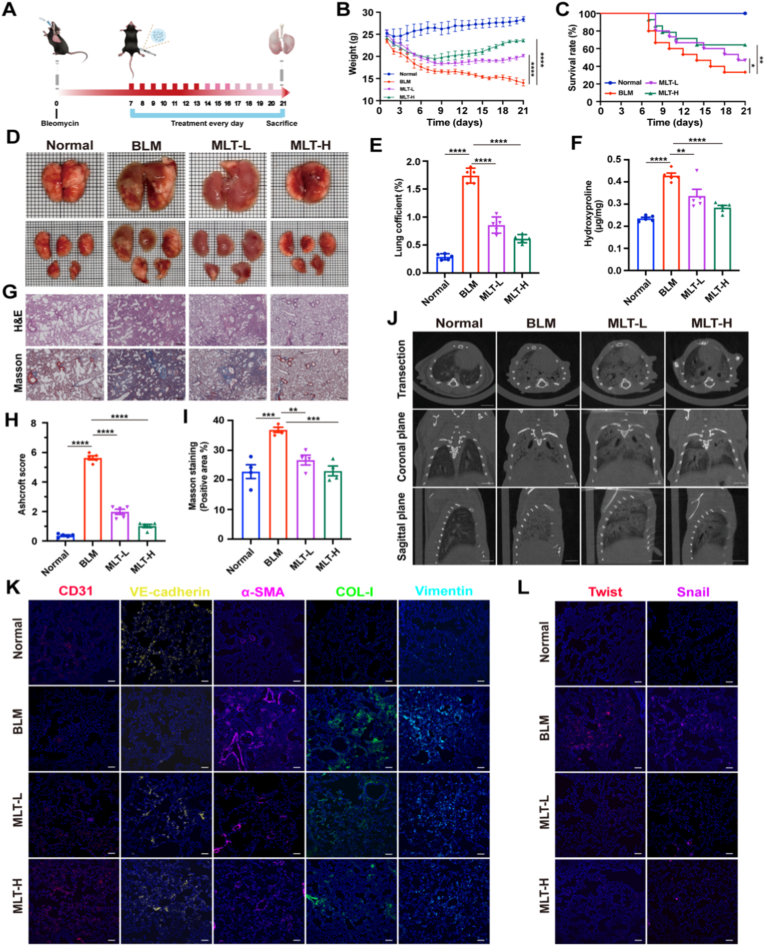
MLT ameliorates BLM-induced PF via EndMT modulation. (A) Schematic diagram of the BLM-induced PF model and treatment regimen. (B) Changes in body weight of mice in each group during the experimental period. (C) Survival rate of mice in each group from modeling to day 21. (D) Representative morphological photographs of lung tissues from each group. (E) Lung coefficient (lung weight/body weight ratio) of mice in each group at the time of euthanasia (n = 5). (F) Hydroxyproline content in lung tissues, reflecting collagen deposition levels (n = 5). (G) Histopathological staining of lung tissues. Scale bar: 20 μm. (H) Quantification of PF by the Ashcroft score (n = 5). (I) Quantitative analysis of Masson staining results, reflecting the relative area of collagen deposition in lung tissues (n = 4). (J) Representative Micro-CT images of lung tissue at three different planes: sagittal, coronal, and transverse sections, showing the overall distribution of pulmonary lesions. Scale bar: 15 mm. (K) Immunofluorescence staining images of key therapeutic indicators in lung tissues. Scale bar: 50 μm. Blue: DAPI; Red: CD31; Yellow: VE-cadherin; Magenta: α-SMA; Green: COL-I; Cyan: Vimentin. (L) Immunofluorescence staining images of nuclear transcription factors Twist and Snail in lung tissues. Scale bar: 50 μm. Blue: DAPI; Red: Twist; Magenta: Snail. Data are presented as the mean ± SEM; ∗*p* < 0.05, ∗∗*p* < 0.01, ∗∗∗*p* < 0.001, ∗∗∗∗*p* < 0.0001.

To further evaluate the effect of MLT on PF, C57BL/6 mice were treated by daily intraperitoneal injection of MLT-L (0.5 mg/kg, low dose) and MLT-H (2 mg/kg, high dose) starting from day 7 after BLM induction (Fig. 5A). As shown in Fig. 5B, mice treated with MLT exhibited sustained weight gain during the later stages of treatment, with the MLT-H group showing body weights comparable to those of the normal control group. Additionally, the survival rate in the BLM model group was 33 %, whereas MLT-L and MLT-H treatments significantly improved survival rates to 47 % and 60 %, respectively (Fig. 5C). Morphological observations indicated that BLM challenge caused severe lung tissue damage. In contrast, MLT treatment significantly attenuated these effects, with the MLT-H group showing more pronounced improvement (Fig. 5D). The lung coefficient, defined as the ratio of lung wet weight to body weight, is a critical physiological parameter for evaluating pulmonary pathological changes in fibrotic models. The BLM group showed a significant increase in lung coefficient compared to the normal control group. However, MLT treatment dose-dependently reversed this elevation, as evidenced by the significantly lower lung coefficient in both MLT-L and MLT-H groups versus the BLM group (Fig. 5E). We further measured hydroxyproline (HYP) content to evaluate collagen deposition in lung tissues. As expected, both MLT-L and MLT-H markedly suppressed the elevated HYP levels (Fig. 5F). Histological analyses further confirmed the anti-fibrotic effects of MLT. H&E staining revealed structural abnormalities in the BLM group, including thickened alveolar walls, collapsed alveolar spaces, prominent collagen deposition, and extensive fibrosis. In contrast, the MLT-H group exhibited a significant reduction in fibrotic areas. Similarly, Masson's trichrome staining demonstrated increased PF in the BLM group compared to the normal controls, whereas the MLT-L and MLT-H groups showed milder fibrosis (Fig. 5G). Next, we assessed fibrosis severity using Ashcroft scoring based on H&E-stained sections. The results revealed that the Ashcroft scores of the MLT-L and MLT-H groups were significantly lower than those of the BLM group (Fig. 5H). Moreover, MLT treatment reduced the collagen deposition area (Fig. 5I). Micro-CT imaging data also showed that the BLM group exhibited extensive high-radiodensity areas, reflecting fibrotic lesions, alveolar collapse, and interstitial thickening (Fig. 5J). However, MLT treatment improved these changes in a dose-dependent manner: MLT-L reduced fibrotic areas partially, whereas MLT-H achieved more substantial recovery, as evidenced by a marked reduction in high-density regions and better preservation of aerated lung parenchyma, consistent with its superior anti-fibrotic efficacy. Collectively, these results demonstrate that MLT intervention alleviates pulmonary edema and abnormal tissue remodeling in fibrotic lungs. Importantly, the therapeutic effects were dose-dependent, with the MLT-H group exhibiting greater improvements across multiple parameters compared to the MLT-L group.

Next, immunofluorescence staining was performed on lung tissues to assess the expression of pivotal markers related to EndMT and fibrosis progression. As shown in Fig. 5K and L, the BLM group exhibited a marked increase in α-SMA, COL-I, and vimentin, suggesting active EndMT and collagen accumulation. Additionally, the nuclear transcription factors Twist and Snail, important regulators of EndMT, were significantly more abundant in the nuclei of the BLM group compared to the normal group. However, MLT treatment attenuated these changes in a dose-dependent manner. Notably, the MLT-H group showed a higher expression of endothelial markers (CD31 and VE-cadherin), and a greater reduction in mesenchymal markers (α-SMA, COL-I, vimentin) and EndMT-related transcription factors (Twist and Snail) compared to both the BLM and MLT-L groups. Collectively, these findings indicate that MLT treatment effectively attenuates BLM-induced PF by inhibiting EndMT in a dose-dependent manner.

### 3.5 M-pLNPs enable prolonged circulation and enhanced lung retention of MLT

**Fig. 6:**
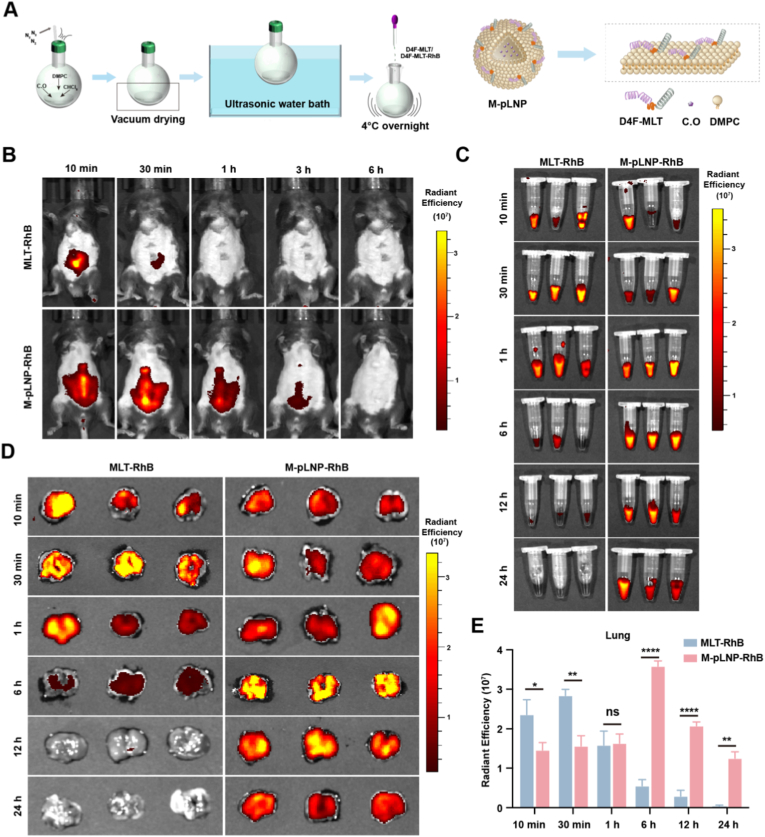
*In vivo* evaluation of M-pLNP for prolonged pulmonary delivery. (A) Schematic diagram illustrating the M-pLNP synthesis process. (B) *In vivo* fluorescence imaging of BLM-induced fibrotic mice at different time points following single intraperitoneal injection of RhB-labeled free MLT or M-pLNPs (15 nmol RhB equivalent per mouse). (C) Fluorescence imaging of blood samples collected at different time points post-injection. (D) Representative fluorescence images of lung tissues from BLM-induced fibrotic mice at indicated time points. (E) Quantitative analysis of mean fluorescence intensity (MFI) of RhB in lung tissues. Data are presented as mean ± SEM (n = 3); ∗*p* < 0.05, ∗∗*p* < 0.01, ∗∗∗∗*p* < 0.0001, ns: not significant.

Given the characteristically short half-life of therapeutic peptides, we next aimed to improve MLT's pharmacokinetic profile through nanoformulation. We therefore employed our previously established peptide-lipid nanoparticle (pLNP) platform, which possesses an extended circulation half-life [32], to enhance MLT's systemic exposure while reducing dosing frequency. The MLT-loaded pLNPs (M-pLNPs) were prepared using the thin-film hydration method (Fig. 6A). To ensure batch-to-batch consistency for the current study, we first characterized the physicochemical properties of the synthesized M-pLNPs. Dynamic light scattering revealed a hydrodynamic diameter of 13.66 ± 4.4 nm, while transmission electron microscopy showed spherical, uniformly dispersed ultra-small nanoparticles (Fig. S6A). The surface potential of M-pLNPs measured 2.35 ± 0.43 mV (Fig. S6B), indicating a nearly neutral surface charge that contributes to the stability of the nanoparticle formulation. To enable fluorescence tracking, the MLT fusion peptide was conjugated with RhB during synthesis and used in the preparation of RhB-labeled M-pLNPs. The successful RhB labeling was confirmed by spectroscopic analysis, showing a characteristic absorption peak at 534 nm and a corresponding fluorescence emission maximum at 570 nm (Fig. S6C and D). Furthermore, the fluorescence intensity exhibited excellent linear correlation with nanoparticle concentration (Fig. S6E), providing a solid basis for reliable quantitative tracking *in vivo*.

We next performed biodistribution studies following intraperitoneal administration to reveal the strikingly different pharmacokinetic profiles between the formulations. Following administration of free MLT, rapid absorption from the peritoneal cavity was evidenced by a substantially weakened signal within 30 min and its subsequent disappearance (Fig. 6B), while a strong fluorescence signal emerged in the systemic circulation by 30 min (Fig. 6C). However, this circulating signal declined abruptly and was nearly undetectable in blood within 6 h, indicating the short systemic half-life of the free MLT peptide. In contrast, M-pLNPs exhibited a superior pharmacokinetic properties. Absorption from the peritoneum was moderately delayed, with complete clearance occurring within approximately 3 h, whereas the blood fluorescence signal increased gradually, reaching a peak around 6 h and maintaining a detectable presence throughout the 24-h observation period (Fig. 6B and C). To quantitatively assess pulmonary retention, lung tissues were collected at various time points for *ex vivo* fluorescence analysis. As shown in Fig. 6D, free MLT exhibited rapid but transient accumulation, peaking at 30 min, followed by a sequential decline that eventually reached near-background levels by 6 h. However, M-pLNPs demonstrated sustained pulmonary retention with detectable fluorescence signals maintained throughout the entire 24-h observation period. Quantitative analysis further confirmed that the fluorescence intensity in lung tissues was markedly higher in the M-pLNP group compared to the free MLT group at the later time points of 6, 12, and 24 h, when the latter showed barely detectable signals (Fig. 6E). Taken together, these results demonstrate that M-pLNPs effectively overcome the pharmacokinetic limitations of native MLT, providing prolonged systemic exposure and enhanced pulmonary retention essential for effective anti-fibrotic therapy.

### 3.6 Nanoparticle-based delivery of MLT potentiates anti-PF efficacy

**Fig. 7:**
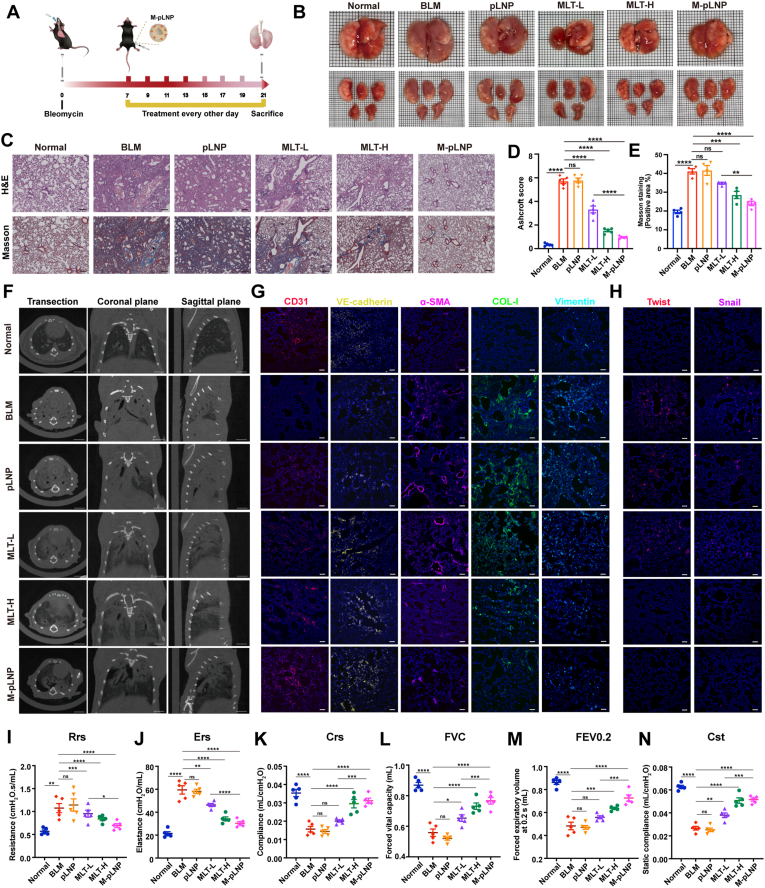
Nanoparticle delivery of MLT potentiates anti-PF efficacy. (A) Experimental timeline of BLM-induced PF and therapeutic intervention. Mice received intratracheal BLM on day 0, followed by intraperitoneal administration of pLNPs, MLT-L (0.5 mg/kg), MLT-H (2 mg/kg), M-pLNPs (0.5 mg/kg as MLT) every other day from day 7 to day 21. (B) Representative macroscopic morphological images of lung tissues from each treatment group. (C) Histopathological analyses of lung tissues. Scale bar: 20 μm. (D) Quantitative analysis of fibrosis severity by Ashcroft scoring. (E) Quantitative assessment of Masson staining. (F) Representative Micro-CT images of lung tissue at three anatomical planes: sagittal, coronal, and transverse, demonstrating the spatial distribution of fibrotic lesions. Scale bar: 15 mm. (G) Immunofluorescence staining of key therapeutic targets in lung tissues. Scale bar: 50 μm. Blue: DAPI; Red: CD31; Yellow: VE-cadherin; Magenta: α-SMA; Green: COL-I; Cyan: Vimentin. (H) Immunofluorescence staining images showing the expression and localization of nuclear transcription factors Twist and Snail in lung tissues. Scale bar: 50 μm. Blue: DAPI; Red: Twist; Magenta: Snail. (I–N) Quantitative analysis of pulmonary function parameters including respiratory system resistance (Rrs) (I), elastance (Ers) (J), compliance (Crs) (K), forced vital capacity (FVC) (L) and forced expiratory volume at 0.2 s (FEV0.2) (M), and static compliance (Cst) (N). Data are presented as the mean ± SEM (n = 5); ∗*p* < 0.05, ∗∗*p* < 0.01, ∗∗∗*p* < 0.001, ∗∗∗∗*p* < 0.0001, ns: not significant.

Based on the promising biodistribution and extended lung retention of M-pLNPs, we next evaluated the anti-fibrotic efficacy of the M-pLNP formulation in comparison with free MLT. Mice were divided into six groups: normal control, BLM-induced model, pLNP, MLT-L (0.5 mg/kg), MLT-H (2 mg/kg), and M-pLNP(0.5 mg/kg as MLT). All treatments were administered via intraperitoneal injection every other day starting from day 7 after PF induction (Fig. 7A). Despite a 75 % reduction in dosage, M-pLNPs administration led to a remarkable restoration of body weight, reaching levels comparable to the normal range (Fig. S7). Survival rate data further underscored the therapeutic superiority of M-pLNPs, with the M-pLNP-treated group exhibiting a 73 % survival rate, compared to 60 % in the MLT-H group and 33 % in the BLM model group (Fig. S8). Similar to the previous experimental protocol, lung tissues from mice in each group were extracted on day 21. A series of morphological and biochemical examinations were performed, including macroscopic observation, H&E staining, Masson staining, lung coefficient measurement, Ashcroft scoring, and HYP content assay. Notably, M-pLNPs treatment not only demonstrated substantial restoration of lung morphology but also more effectively reduced lung coefficient and HYP levels compared to MLT-L group (Fig. 7B–S9 and S10), indicating its superior efficacy in mitigating fibrotic progression. Histological analyses consistently revealed its superior antifibrotic efficacy, with significantly reduced fibrotic areas, near-normal alveolar architecture, and markedly attenuated collagen deposition (Fig. 7C–E). Non-invasive Micro-CT imaging showed that M-pLNPs treatment resulted in a more pronounced reduction of high-density fibrotic regions and better preservation of aerated lung parenchyma compared to MLT-L (Fig. 7F). In contrast, the pLNPs vehicle control group showed no significant improvement in fibrotic areas, with a pattern indistinguishable from the BLM model group, confirming that the therapeutic benefits were specifically mediated by MLT rather than the nanoparticle carrier itself. Remarkably, the therapeutic effect of M-pLNPs was comparable to that of the high-dose MLT-H group, despite a 75 % reduction in dosage.

Beyond these structural improvements, we further investigated the molecular mechanisms underlying the anti-fibrotic effects. In both the BLM and pLNPs groups, characteristic EndMT progression was observed, marked by downregulation of CD31 and VE-cadherin alongside upregulation of α-SMA, COL-I, and vimentin (Fig. 7G). Importantly, M-pLNPs more effectively reversed these changes by enhancing endothelial integrity through upregulation of CD31 and VE-cadherin, and suppressing mesenchymal transition via downregulation of α-SMA, COL-I, and vimentin, compared to the same dose of free MLT-L. Furthermore, M-pLNPs significantly inhibited the nuclear translocation of Twist and Snail (Fig. 7H). Thus, immunofluorescence analysis demonstrated the superior efficacy of M-pLNPs in modulating EndMT markers compared to MLT-L, achieving a level comparable to the MLT-H group. Finally, pulmonary function tests was performed on day 21 to evaluate the therapeutic effects of M-pLNPs on lung function compared with free MLT. Elevated respiratory system resistance (Rrs) and elastance (Ers) are characteristic hallmarks of PF, reflecting compromised lung contractility and increased elastic stiffness in BLM-injured lungs. As shown in Fig. 7I and J, both parameters were significantly improved by M-pLNPs treatment. Consistent with this improvement, other key pulmonary function metrics, including compliance (Crs), forced vital capacity (FVC), forced expiratory volume at 0.2 s (FEV0.2), and static compliance (Cst), were also significantly ameliorated by M-pLNPs treatment, particularly Crs and FVC (Fig. 7K–N). Collectively, our results demonstrate that nanoparticle-based delivery significantly enhances the anti-fibrotic efficacy of MLT, enabling robust therapeutic outcomes at a substantially reduced dose.

## 4 Discussion

Despite advances in treatment strategies for PF, the prognosis remains unsatisfactory. Only two drugs, nintedanib and pirfenidone, have been approved for conditional recommendation for PF, both exerting anti-fibrotic effects primarily through anti-inflammatory mechanisms [33]. While vascular endothelial dysfunction, particularly EndMT, is increasingly implicated in PF pathogenesis, targeted interventions remain scarce. In this study, we demonstrate MLT specifically targets the overexpressed NRP-1 receptor in fibrotic tissue, implementing dual inhibition of the TGF-β/Smad and MAPK pathways and ultimately blocking EndMT in PF. Furthermore, encapsulation of MLT into peptide-lipid nanoparticles moderately slows its systemic absorption while markedly prolonging its pulmonary retention compared to the free MLT. This optimized biodistribution profile enables effective anti-fibrotic efficacy while reducing administration frequency, demonstrating a favorable therapeutic profile with potency comparable to high-dose free MLT. These findings establish NRP-1-directed EndMT inhibition as a therapeutically viable strategy for PF and identify MLT as a novel anti-fibrotic agent that targets this specific pathological process.

NRP-1 is now recognized as a central regulator in the pathogenesis of PF, primarily through its function as a critical co-receptor for TGF-β that amplifies TGF-β/Smad signaling and drives EndMT. The pro-fibrotic function of NRP-1 is further supported by recent therapeutic study demonstrating that pharmacological inhibition of NRP-1 with the specific inhibitor A7R peptide significantly attenuates TGF-β/Smad signaling and exhibits considerable anti-fibrotic efficacy [34]. Notably, the expression profile of NRP-1 in fibrotic lungs is not restricted to endothelial cells. Emerging evidence has identified NRP-1 as a specific marker for group 2 innate lymphoid cells (ILC2s) in lung tissue, where TGF-β induced NRP-1 expression potentiates ILC2 function and type 2 immunity by upregulating the IL-33 receptor ST2 [35]. In addition, NRP-1 expression was detected across all macrophage subsets in the lung microenvironment, with significantly higher frequency of NRP-1-positive alveolar macrophages (AMs) in fibrotic lung tissues compared to normal controls [36]. Importantly, macrophage polarization plays a critical role in the pathogenesis of PF, with M1 macrophages contributing to initial inflammatory damage and M2 macrophages driving subsequent fibrotic progression through extracellular matrix deposition [37]. Given that MLT has been reported to suppress pro-inflammatory cytokines and modulate NF-κB and MAPK signaling [38], and has been observed to selectively reduce M2-like macrophage populations without significant cytotoxicity *in vitro* [39], the therapeutic benefits observed in our study likely involve a multi-faceted mechanism. This encompasses not only direct suppression of EndMT but also modulation of immune responses through NRP-1-expressing cells such as ILC2s and macrophages.

The R/KXXR/K sequence motif serves as a well-established structural determinant for high-affinity binding to NRP-1 and subsequent induction of cellular internalization and tissue penetration [40]. This motif is inactive unless it occupies the C-terminal position in the peptide. Notably, internal R/KXXR/K motifs can remain cryptic until specific proteolytic cleavage exposes a functional C-terminal motif capable of engaging NRP-1 [28]. This mechanism is well illustrated by several engineered peptides, as demonstrated by the tumor-homing peptide iRGD which initially binds αV integrins through its RGD motif before proteolytic cleavage unveils its C-terminal CendR motif, thereby facilitating NRP-1-dependent tissue penetration [41]. Similarly, a PL3-derived linear peptide achieves precise NRP-1 targeting exclusively through uPA-mediated proteolytic cleavage, effectively minimizing off-target accumulation while maintaining tumor penetration capability [42]. In the present study, we observed that MLT possesses an internal R/KXXR/K motif yet demonstrates substantial NRP-1 binding affinity. Molecular docking analyses and cellular competitive inhibition assays consistently support this interaction. Given that MLT's R/KXXR/K sequence is not natively C-terminal, its observed NRP-1 targeting ability suggests a potential activation mechanism involving proteolytic processing. We hypothesize that MLT may be hydrolyzed by cell membrane-associated or extracellular proteases, thereby exposing its cryptic R/KXXR/K motif at a newly formed C-terminus and enabling productive binding to NRP-1. This model is consistent with recent studies demonstrating that protease-mediated C-terminal switching can activate internal R/KXXR/K motifs in other peptide systems, underscoring the broader relevance of this regulatory mechanism in modulating NRP-1 interactions.

MLT, as the main active component of bee venom, exhibits hydrophobic and amphipathic properties with nonspecific cytolytic activity [43]. High enough concentrations make it toxic to both cells and tissues. Based on several studies, intraperitoneal injection of safe doses of MLT (0.1–5 mg/kg) in treated animal models appears to be safe with no significant adverse effects on central nervous, cardiovascular, respiratory, and gastrointestinal function [39]. To avoid the cytotoxicity of MLT, in this study, we selected low-dose MLT (0.5 mg/kg) and high-dose MLT (2 mg/kg) within the safe dose range of MLT, and through multiple intraperitoneal administration, we showed excellent PF treatment effect. However, intraperitoneal injection of MLT has the disadvantages of short metabolic cycles and many administration times, while intravenous injection of MLT has erythrocytic toxicity. To overcome these shortcomings, MLT can be conjured with other molecules, such as alpha-helical peptide [44], perfluorocarbon [45], manganese, etc., to formulate nanoparticles that enhance drug stability, control drug release, reduce administration frequency, and minimize drug toxicity. Although our study demonstrates that pLNP-mediated delivery of MLT significantly prolongs pulmonary retention and slows systemic absorption, we also observed notable accumulation in the brain and liver. This off-target distribution may be attributed to the endogenous expression of NRP-1 in these tissues, potentially facilitating unintended MLT uptake. Importantly, MLT's inherent targeting capability toward NRP-1 upregulated in fibrotic lungs remains the primary mechanism for its pulmonary accumulation. Two strategic approaches warrant further investigation to optimize delivery specificity. One involves refining the nanoparticle design through incorporation of tissue-specific targeting elements or microenvironment-responsive components to enhance pulmonary selectivity at the molecular level, while the other employs aerosolized inhalation as an alternative delivery route to maximize local lung deposition.

In summary, MLT ameliorates PF through high-affinity binding to the NRP-1 co-receptor, disrupting its synergistic function with TGF-β receptors and consequently suppressing downstream Smad2/3 phosphorylation and MAPK pathway activation. Importantly, nano-encapsulation enhances the therapeutic efficacy of MLT at a reduced dose through prolonged lung retention. This study thereby establishes NRP-1 co-receptor interception coupled with advanced nano-delivery as a transformative paradigm for precision anti-fibrotic therapy.

## CRediT authorship contribution statement

**Ming Hu:** Writing – original draft, Investigation, Conceptualization. **Yingying Wan:** Writing – original draft, Investigation. **Jiakang Chen:** Methodology, Investigation. **Chengwei Zhang:** Writing – review & editing, Methodology, Formal analysis. **Shuze Li:** Writing – review & editing, Methodology, Investigation. **Bingbing Shan:** Methodology. **Ling Wu:** Writing – review & editing, Writing – original draft, Supervision, Conceptualization. **Xiang Yu:** Writing – review & editing, Writing – original draft, Supervision, Project administration, Conceptualization.

## Declaration of competing interest

The authors declare that they have no known competing financial interests or personal relationships that could have appeared to influence the work reported in this paper.

## Data Availability

Data will be made available on request.

## References

[1] Podolanczuk A.J., Thomson C.C., Remy-Jardin M., Richeldi L., Martinez F.J., Kolb M., Raghu G. (2023). Idiopathic pulmonary fibrosis: state of the art for 2023. Eur. Respir. J..

[2] Chanda D., Otoupalova E., Smith S.R., Volckaert T., De Langhe S.P., Thannickal V.J. (2019). Developmental pathways in the pathogenesis of lung fibrosis. Mol Aspects Med.

[3] Sohal S.S. (2023). Endothelial to mesenchymal transition: a novel pathological feature of pulmonary fibrosis. Eur. Respir. J..

[4] Hashimoto N., Phan S.H., Imaizumi K., Matsuo M., Nakashima H., Kawabe T., Shimokata K., Hasegawa Y. (2010). Endothelial-mesenchymal transition in bleomycin-induced pulmonary fibrosis. Am. J. Respir. Cell Mol. Biol..

[5] Zeisberg E.M., Tarnavski O., Zeisberg M., Dorfman A.L., McMullen J.R., Gustafsson E., Chandraker A., Yuan X., Pu W.T., Roberts A.B., Neilson E.G., Sayegh M.H., Izumo S., Kalluri R. (2007). Endothelial-to-mesenchymal transition contributes to cardiac fibrosis. Nat Med.

[6] Pardali E., Sanchez-Duffhues G., Gomez-Puerto M.C., Ten Dijke P. (2017). TGF-β-Induced endothelial-mesenchymal transition in fibrotic diseases. Int. J. Mol. Sci..

[7] Györfi A.H., Matei A.E., Distler J.H.W. (2018). Targeting TGF-β signaling for the treatment of fibrosis. Matrix Biol..

[8] Hau P., Jachimczak P., Bogdahn U. (2009). Treatment of malignant gliomas with TGF-beta2 antisense oligonucleotides. Expert Rev. Anticancer Ther..

[9] Kelly R.J., Morris J.C. (2010). Transforming growth factor-beta: a target for cancer therapy. J Immunotoxicol.

[10] Li Y., Wang Z., Xu H., Hong Y., Shi M., Hu B., Wang X., Ma S., Wang M., Cao C., Zhu H., Hu D., Xu C., Lin Y., Xu G., Yao Y., Zeng R. (2024). Targeting the transmembrane cytokine co-receptor neuropilin-1 in distal tubules improves renal injury and fibrosis. Nat. Commun..

[11] Hong Q., Kim H., Cai G.Y., Chen X.M., He J.C., Lee K. (2025). Modulation of TGF-β signaling new approaches toward kidney disease and fibrosis therapy. Int. J. Biol. Sci..

[12] Nickel J., Ten Dijke P., Mueller T.D. (2018). TGF-β family co-receptor function and signaling. Acta Biochim. Biophys. Sin..

[13] Chen W., Ten Dijke P. (2016). Immunoregulation by members of the TGFβ superfamily. Nat. Rev. Immunol..

[14] Glinka Y., Prud'homme G.J. (2008). Neuropilin-1 is a receptor for transforming growth factor beta-1, activates its latent form, and promotes regulatory T cell activity. J. Leukoc. Biol..

[15] Glinka Y., Stoilova S., Mohammed N., Prud'homme G.J. (2011). Neuropilin-1 exerts co-receptor function for TGF-beta-1 on the membrane of cancer cells and enhances responses to both latent and active TGF-beta. Carcinogenesis.

[16] Cao Y., Szabolcs A., Dutta S.K., Yaqoob U., Jagavelu K., Wang L., Leof E.B., Urrutia R.A., Shah V.H., Mukhopadhyay D. (2010). Neuropilin-1 mediates divergent R-Smad signaling and the myofibroblast phenotype. J. Biol. Chem..

[17] Kwiatkowski S.C., Guerrero P.A., Hirota S., Chen Z., Morales J.E., Aghi M., McCarty J.H. (2017). Neuropilin-1 modulates TGFβ signaling to drive glioblastoma growth and recurrence after anti-angiogenic therapy. PLoS One.

[18] Cao S., Yaqoob U., Das A., Shergill U., Jagavelu K., Huebert R.C., Routray C., Abdelmoneim S., Vasdev M., Leof E., Charlton M., Watts R.J., Mukhopadhyay D., Shah V.H. (2010). Neuropilin-1 promotes cirrhosis of the rodent and human liver by enhancing PDGF/TGF-beta signaling in hepatic stellate cells. J. Clin. Investig..

[19] Oršolić N. (2012). Bee venom in cancer therapy. Cancer Metastasis Rev..

[20] Roberto de Oliveira M. (2025). Melittin-induced modulation of mitochondrial physiology: beyond the antitumoral actions. Toxicon.

[21] Yu X., Jia S., Yu S., Chen Y., Zhang C., Chen H., Dai Y. (2023). Recent advances in melittin-based nanoparticles for antitumor treatment: from mechanisms to targeted delivery strategies. J. Nanobiotechnol..

[22] Paray B.A., Ahmad A., Khan J.M., Taufiq F., Pathan A., Malik A., Ahmed M.Z. (2021). The role of the multifunctional antimicrobial peptide melittin in gene delivery. Drug Discov. Today.

[23] Jia S., Chen Y., Zhuo C., Hu M., Zhang C., Cai H., Li X., Chen H., Yu X. (2025). Aptamer-modified melittin micelles efficiently inhibit osteosarcoma deterioration by inducing immunogenic cell death. Colloids Surf. B Biointerfaces.

[24] Huang J.Y., Peng S.F., Chueh F.S., Chen P.Y., Huang Y.P., Huang W.W., Chung J.G. (2021). Melittin suppresses epithelial-mesenchymal transition and metastasis in human gastric cancer AGS cells via regulating Wnt/BMP associated pathway. Biosci. Biotechnol. Biochem..

[25] Jeong Y.J., Park Y.Y., Park K.K., Choi Y.H., Kim C.H., Chang Y.C. (2019). Bee venom suppresses EGF-Induced epithelial-mesenchymal transition and tumor invasion in lung cancer cells. Am. J. Chin. Med..

[26] Wang X., Li H., Lu X., Wen C., Huo Z., Shi M., Tang X., Chen H., Peng C., Fang Y., Deng X., Shen B. (2018). Melittin-induced long non-coding RNA NONHSAT105177 inhibits proliferation and migration of pancreatic ductal adenocarcinoma. Cell Death Dis..

[27] Park S.H., Cho H.J., Jeong Y.J., Shin J.M., Kang J.H., Park K.K., Choe J.Y., Park Y.Y., Bae Y.S., Han S.M., Moon S.K., Kim W.J., Choi Y.H., Chang Y.C. (2014). Melittin inhibits TGF-β-induced pro-fibrotic gene expression through the suppression of the TGFβRII-Smad, ERK1/2 and JNK-mediated signaling pathway. Am. J. Chin. Med..

[28] Teesalu T., Sugahara K.N., Kotamraju V.R., Ruoslahti E. (2009). C-end rule peptides mediate neuropilin-1-dependent cell, vascular, and tissue penetration. Proc. Natl. Acad. Sci. U. S. A..

[29] Ruoslahti E. (2012). Peptides as targeting elements and tissue penetration devices for nanoparticles. Adv Mater.

[30] Matkar P.N., Singh K.K., Rudenko D., Kim Y.J., Kuliszewski M.A., Prud'homme G.J., Hedley D.W., Leong-Poi H. (2016). Novel regulatory role of neuropilin-1 in endothelial-to-mesenchymal transition and fibrosis in pancreatic ductal adenocarcinoma. Oncotarget.

[31] Wu M.H., Chen Y.L., Lee K.H., Chang C.C., Cheng T.M., Wu S.Y., Tu C.C., Tsui W.L. (2017). Glycosylation-dependent galectin-1/neuropilin-1 interactions promote liver fibrosis through activation of TGF-β- and PDGF-like signals in hepatic stellate cells. Sci. Rep..

[32] Huang C., Jin H., Qian Y., Qi S., Luo H., Luo Q., Zhang Z. (2013). Hybrid melittin cytolytic peptide-driven ultrasmall lipid nanoparticles block melanoma growth in vivo. ACS Nano.

[33] Noble P.W., Barkauskas C.E., Jiang D. (2012). Pulmonary fibrosis: patterns and perpetrators. J. Clin. Investig..

[34] Pulivendala G., Bale S., Yanala S.K., Sangaraju R., Godugu C. (2025). Inhibiting Neuropilin-1 as a novel therapeutic approach to mitigate pulmonary fibrosis: highlighting the potential anti-fibrotic effects of ATWLPPR peptide. Int. Immunopharmacol..

[35] Zhang J., Qiu J., Zhou W., Cao J., Hu X., Mi W., Su B., He B., Qiu J., Shen L. (2022). Neuropilin-1 mediates lung tissue-specific control of ILC2 function in type 2 immunity. Nat. Immunol..

[36] Aung N.Y., Ohe R., Meng H., Kabasawa T., Yang S., Kato T., Yamakawa M. (2016). Specific neuropilins expression in alveolar macrophages among tissue-specific macrophages. PLoS One.

[37] Ge Z., Chen Y., Ma L., Hu F., Xie L. (2024). Macrophage polarization and its impact on idiopathic pulmonary fibrosis. Front. Immunol..

[38] Lee G., Bae H. (2016). Anti-inflammatory applications of melittin, a major component of Bee venom: detailed mechanism of action and adverse effects. Molecules.

[39] Fan X.G., Pei S.Y., Zhou D., Zhou P.C., Huang Y., Hu X.W., Li T., Wang Y., Huang Z.B., Li N. (2021). Melittin ameliorates inflammation in mouse acute liver failure via inhibition of PKM2-mediated Warburg effect. Acta Pharmacol. Sin..

[40] Sugahara K.N., Teesalu T., Karmali P.P., Kotamraju V.R., Agemy L., Greenwald D.R., Ruoslahti E. (2010). Coadministration of a tumor-penetrating peptide enhances the efficacy of cancer drugs. Science.

[41] Sugahara K.N., Braun G.B., de Mendoza T.H., Kotamraju V.R., French R.P., Lowy A.M., Teesalu T., Ruoslahti E. (2015). Tumor-penetrating iRGD peptide inhibits metastasis. Mol Cancer Ther.

[42] Tobi A., Haugas M., Rabi K., Sethi J., Põšnograjeva K., Paiste P., Jagomäe T., Pleiko K., Lingasamy P., Teesalu T. (2024). Protease-activated CendR peptides targeting tenascin-C: mitigating off-target tissue accumulation. Drug Deliv Transl Res.

[43] Carpena M., Nuñez-Estevez B., Soria-Lopez A., Simal-Gandara J. (2020). Bee venom: an updating review of its bioactive molecules and its health applications. Nutrients.

[44] Yu X., Dai Y., Zhao Y., Qi S., Liu L., Lu L., Luo Q., Zhang Z. (2020). Melittin-lipid nanoparticles target to lymph nodes and elicit a systemic anti-tumor immune response. Nat. Commun..

[45] Soman N.R., Lanza G.M., Heuser J.M., Schlesinger P.H., Wickline S.A. (2008). Synthesis and characterization of stable fluorocarbon nanostructures as drug delivery vehicles for cytolytic peptides. Nano Lett..

