# Supplementary material for: Modulation of endothelial-to-mesenchymal transition via NRP-1 targeting with melittin attenuates pulmonary fibrosis
Source: Mater Today Bio. 2025 Dec 9;36:102659. doi: 10.1016/j.mtbio.2025.102659 (PMC12794516; doi:10.1016/j.mtbio.2025.102659)
Supplement: Multimedia component 1 [file mmc1.docx]

**Modulation of endothelial-to-mesenchymal transition via NRP-1 targeting with melittin attenuates pulmonary fibrosis**

Ming Hu^a,b,1^, Yingying Wan^c,1^, Jiakang Chen^a,b,1^, Chengwei Zhang^c^, Shuze Li^a,b^, Bingbing Shan^a,b^, Ling Wu^d,*^, Xiang Yu ^a,b,**^

^a^ State Key Laboratory of Digital Medical Engineering, School of Biomedical Engineering, Hainan University

^b^ Key Laboratory of Biomedical Engineering of Hainan Province, One Health Institute, Hainan University

^c^ Hubei Key Laboratory of Tumor Microenvironment and Immunotherapy, China Three Gorges University

^d^ Sanya Yazhou Bay Science and Technology City Hospital, Sanya, China

^*^Corresponding author

^**^Corresponding author

E-mail addresses: [wuling@ctgu.edu.cn](mailto:wuling@ctgu.edu.cn) (L, Wu), [yuxiang@hainanu.edu.cn](mailto:yuxiang@hainanu.edu.cn) (X, Yu).

^1^These authors contributed equally to this work

**Supplementary Material**

**Table. S1**. PCR primers used in this study

| Primer | Sequence (5'→3') |
| --- | --- |
| CD31 forward  CD31 reverse | ACAGGACCGCGTTTTATCCTT  CCTTCCCAGTTCTGGGTTCTT |
| VE-cadherin forward  VE-cadherin reverse | GTTCACCTTCTGCGAGGATATG  GATGGTGAGGATGCAGAGTAAG |
| α-SMA forward  α-SMA reverse | ACCCAGCACCATGAAGATCA  TTTGCGGTGGACAATGGAAG |
| Vimentin forward  Vimentin reverse | TACACAATTGCCTCTCCCCC  ACTCCTGTCTGAGATTACCCT |
| β-actin forward  β-actin reverse | GGACTTCGAGCAAGAGATGG  AGCACTGTGTTGGCGTACAG |


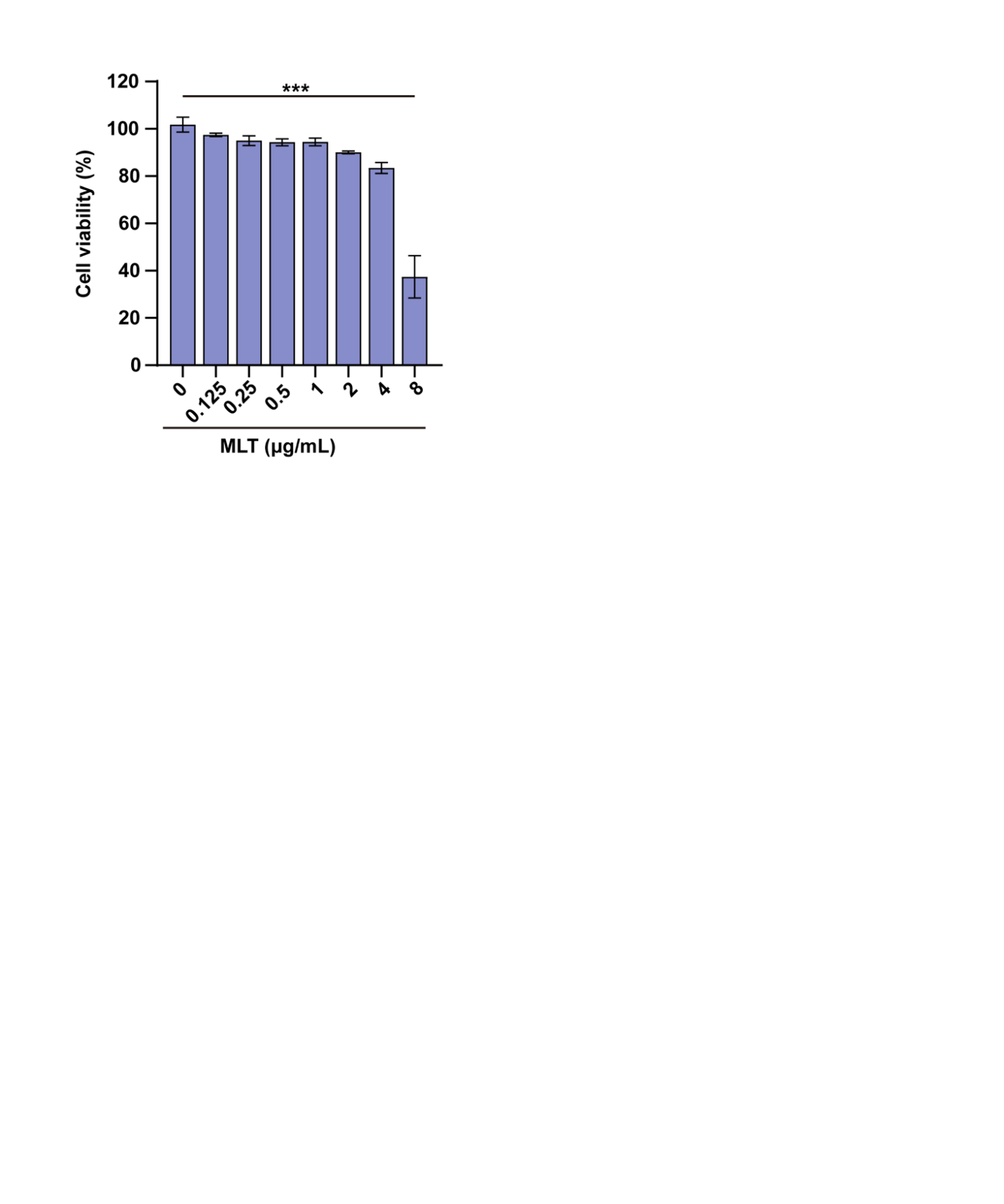


**Fig. S1**. Cell viability of HUVECs treated with MLT was determined by the MTS assay. Data are presented as the mean ± SEM (n = 3); ****p* < 0.001.


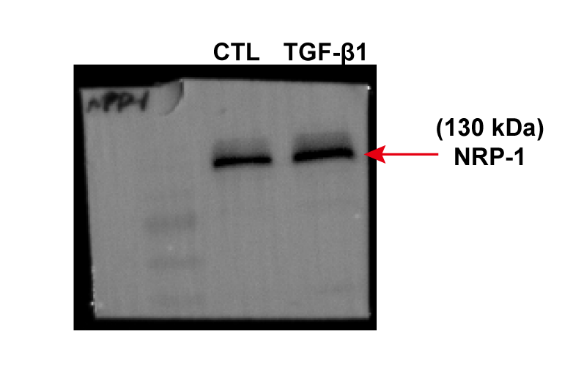


**Fig. S2**. The original western blot image of Figure 1G. Red arrow indicates the protein of interest (NRP-1) and its molecular weight.


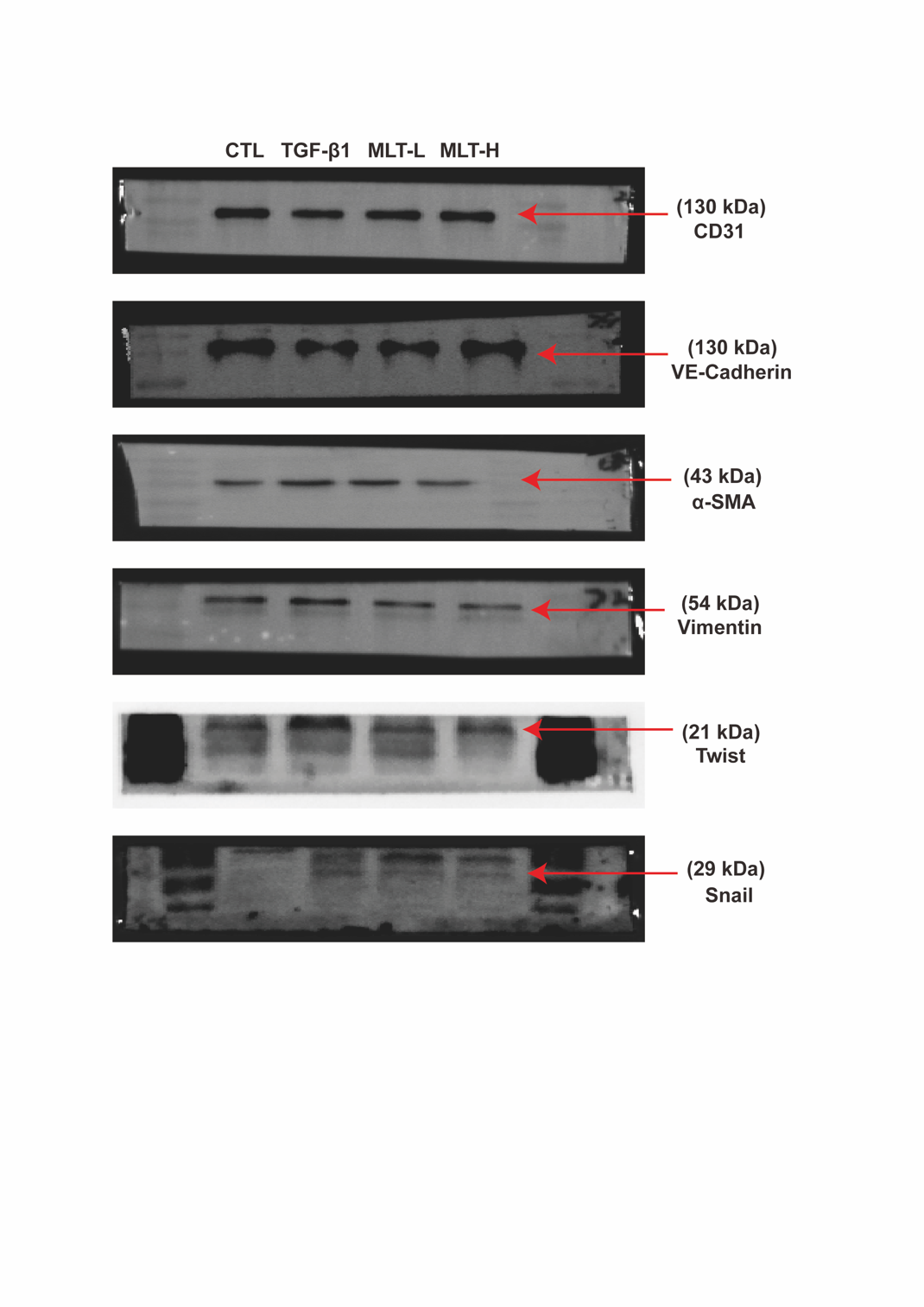


**Fig. S3**. The original western blot images of Figure 3E. The proteins of interest (CD31, VE-cadherin, α-SMA, Vimentin, Twist, Snail) are indicated by red arrows, with their respective molecular weights.


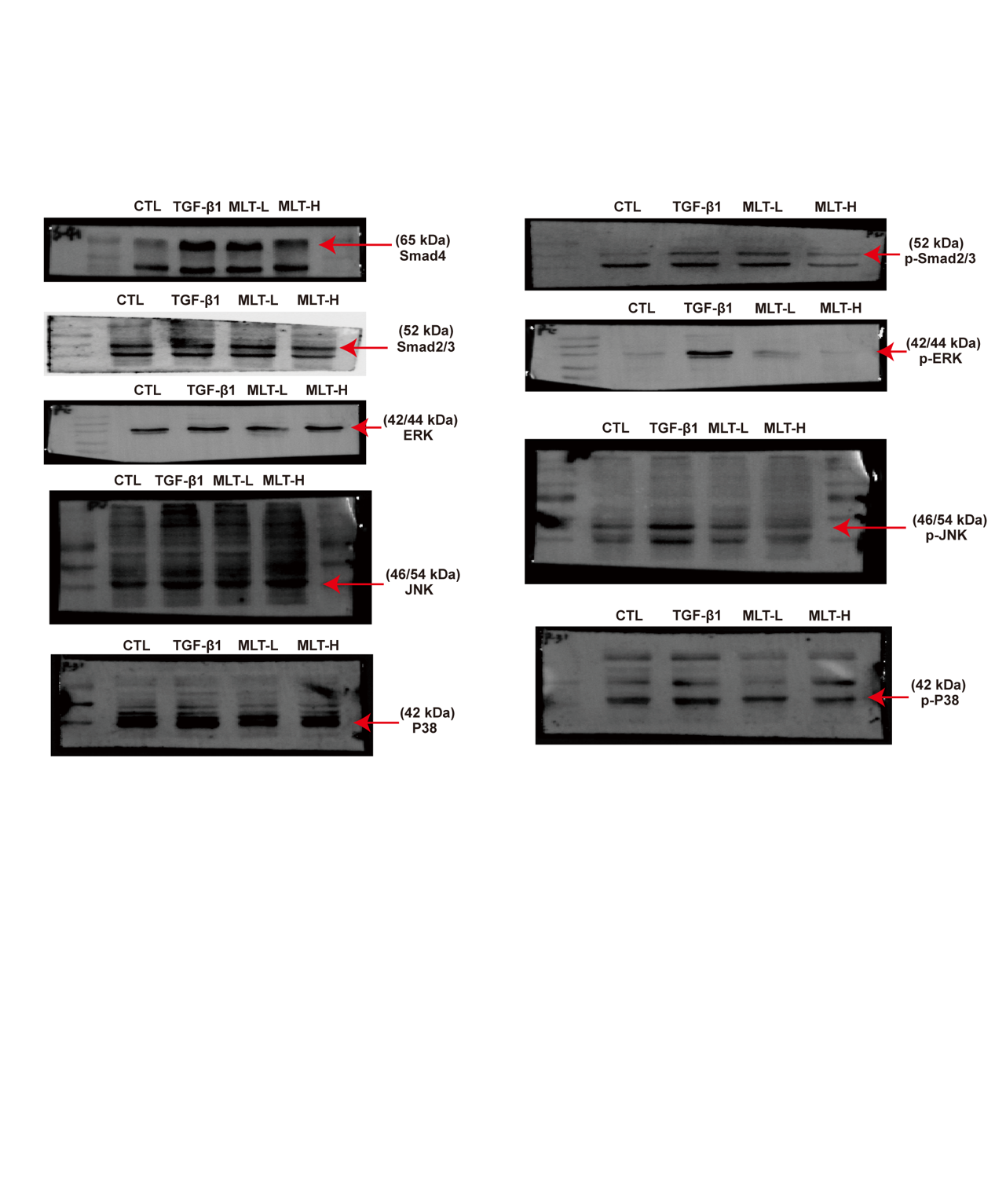


**Fig. S4**. The original western blot images of Figure 3N. The proteins of interest (Smad4, Smad2/3, ERK, JNK, P38, and their phosphorylated forms) are indicated by red arrows, with their respective molecular weights.


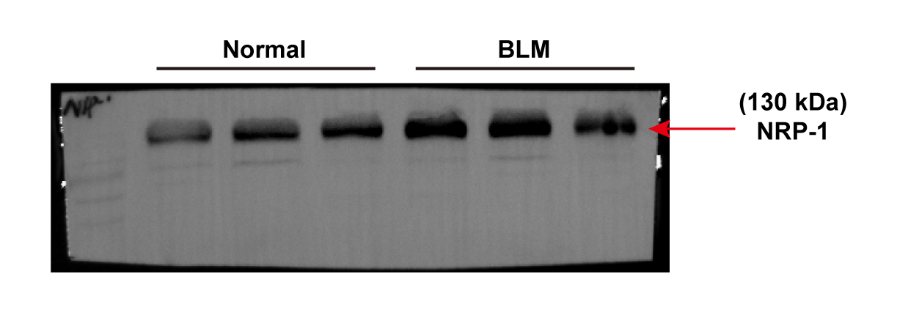


**Fig. S5**. The original western blot image of Figure 4A. Red arrow indicates the protein of interest (NRP-1) and its molecular weight.


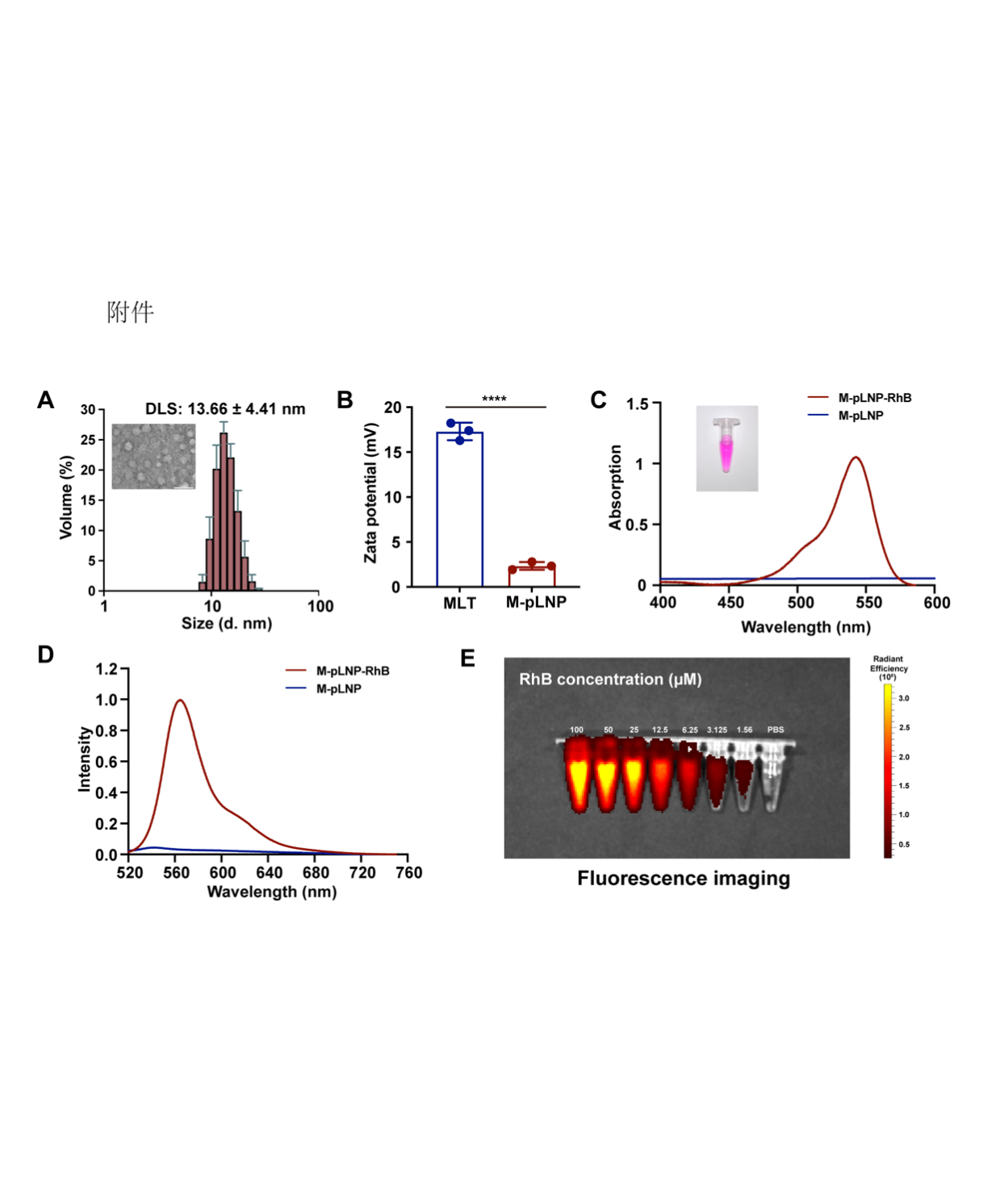


**Fig. S6**. **Characterization of M-pLNPs.** (A) Average particle sizes of M-pLNPs. (B) Zeta potential of MLT and M-pLNPs by DLS. (C) Ultraviolet-visible spectra of M-pLNPs and M-pLNP-RhB. (D) Fluorescence spectra of M-pLNPs and M-pLNP-RhB. (E) Fluorescence imaging of M-pLNP-RhB at different RhB concentrations (100, 50, 25, 12.5, 6.25, 3.125 and 1.56 μM). Data are presented as the mean ± SEM (n = 5); *****p* < 0.0001.


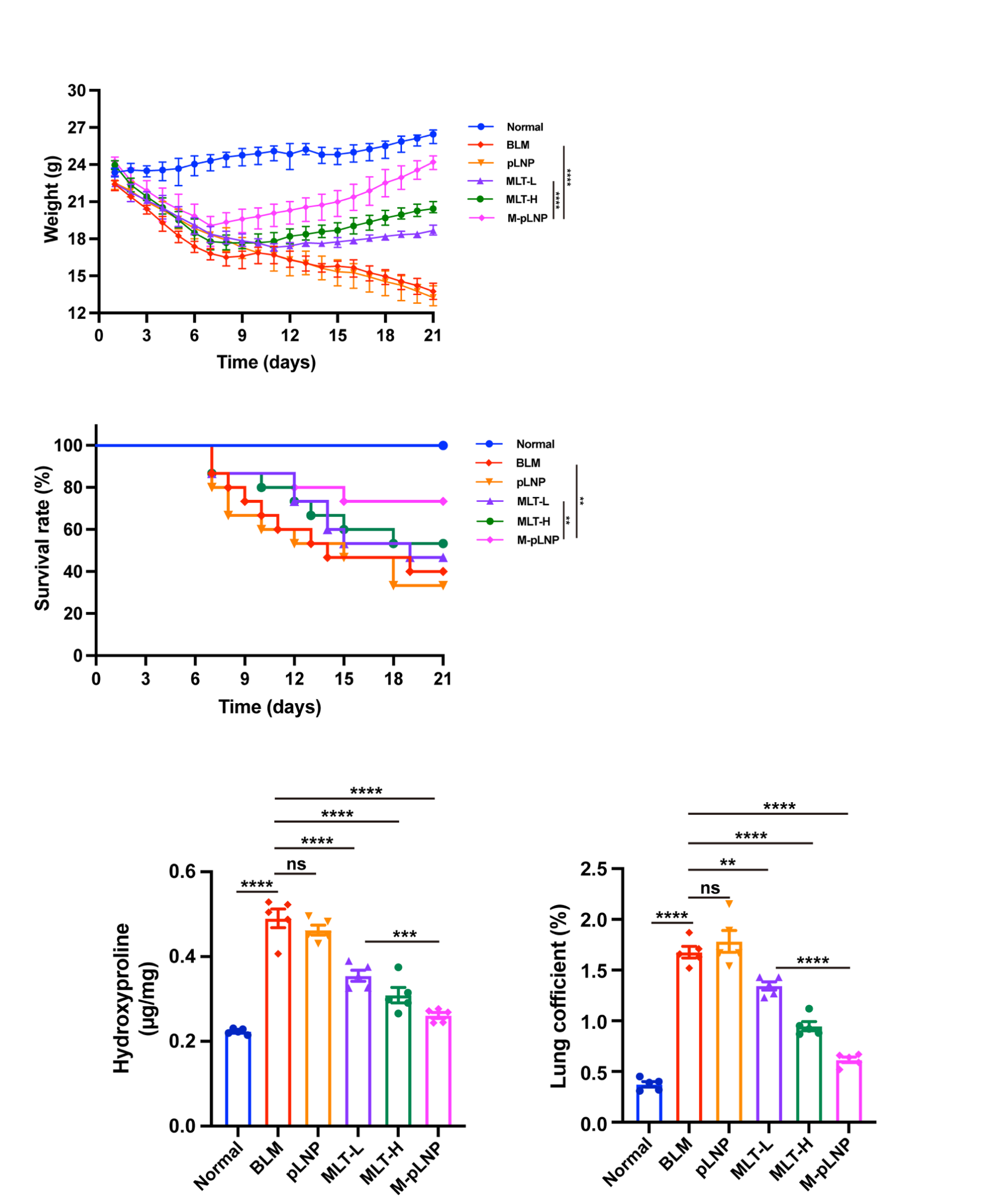


**Fig. S7**. Dynamic changes in body weight among different experimental groups throughout the study period. Data are presented as the mean ± SEM (n = 5); *****p* < 0.0001.


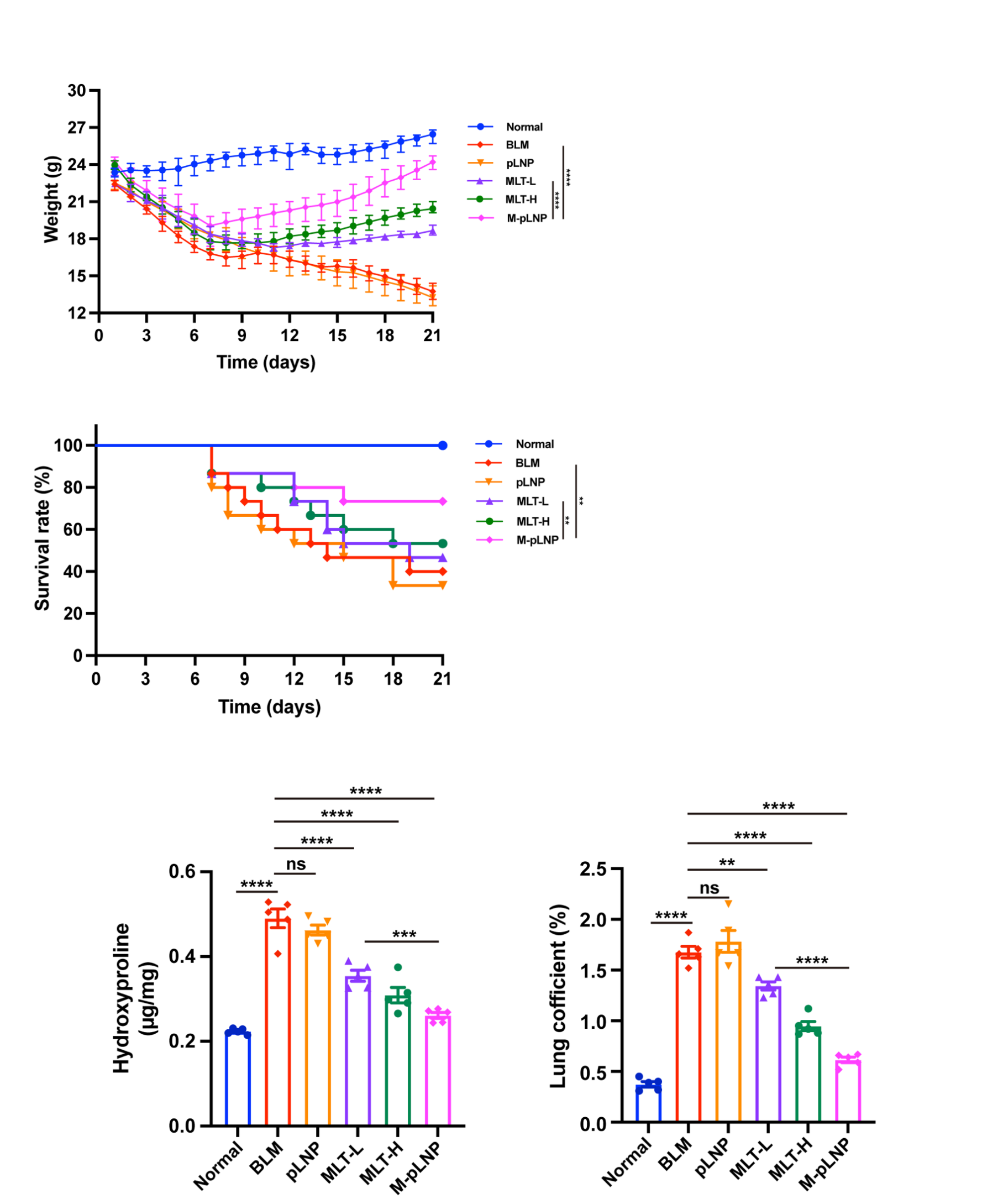


**Fig. S8**. Survival curves showing the survival rate of mice in each group from modeling initiation to day 21. The difference in survival between groups was compared by Log-rank test (n = 5). ***p* < 0.01.


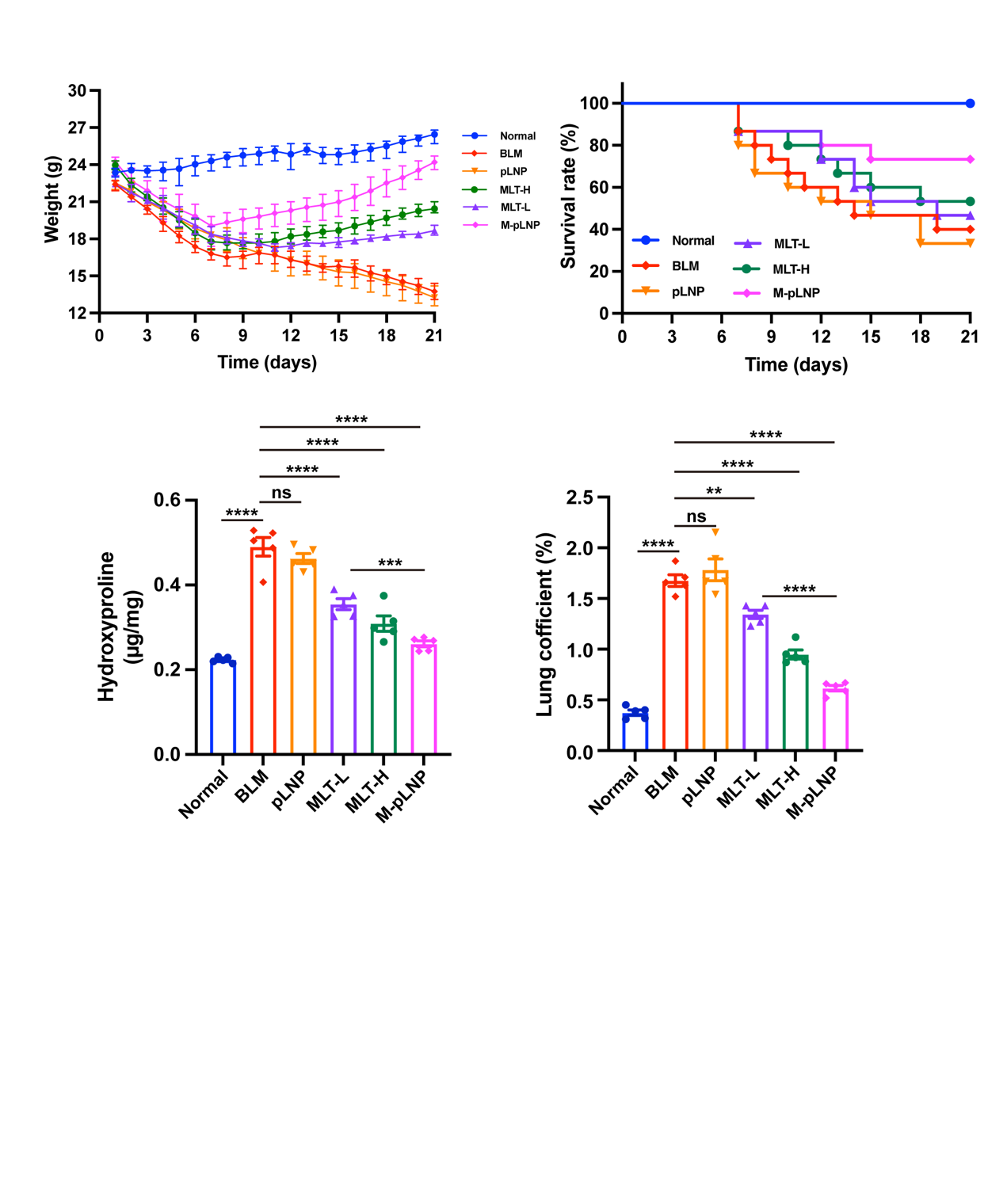


**Fig. S9**. Lung coefficient, calculated as the ratio of lung weight to body weight, measured at the time of euthanasia. Data are presented as the mean ± SEM (n = 5); ns: not significant, ***p* < 0.01, *****p* < 0.0001.


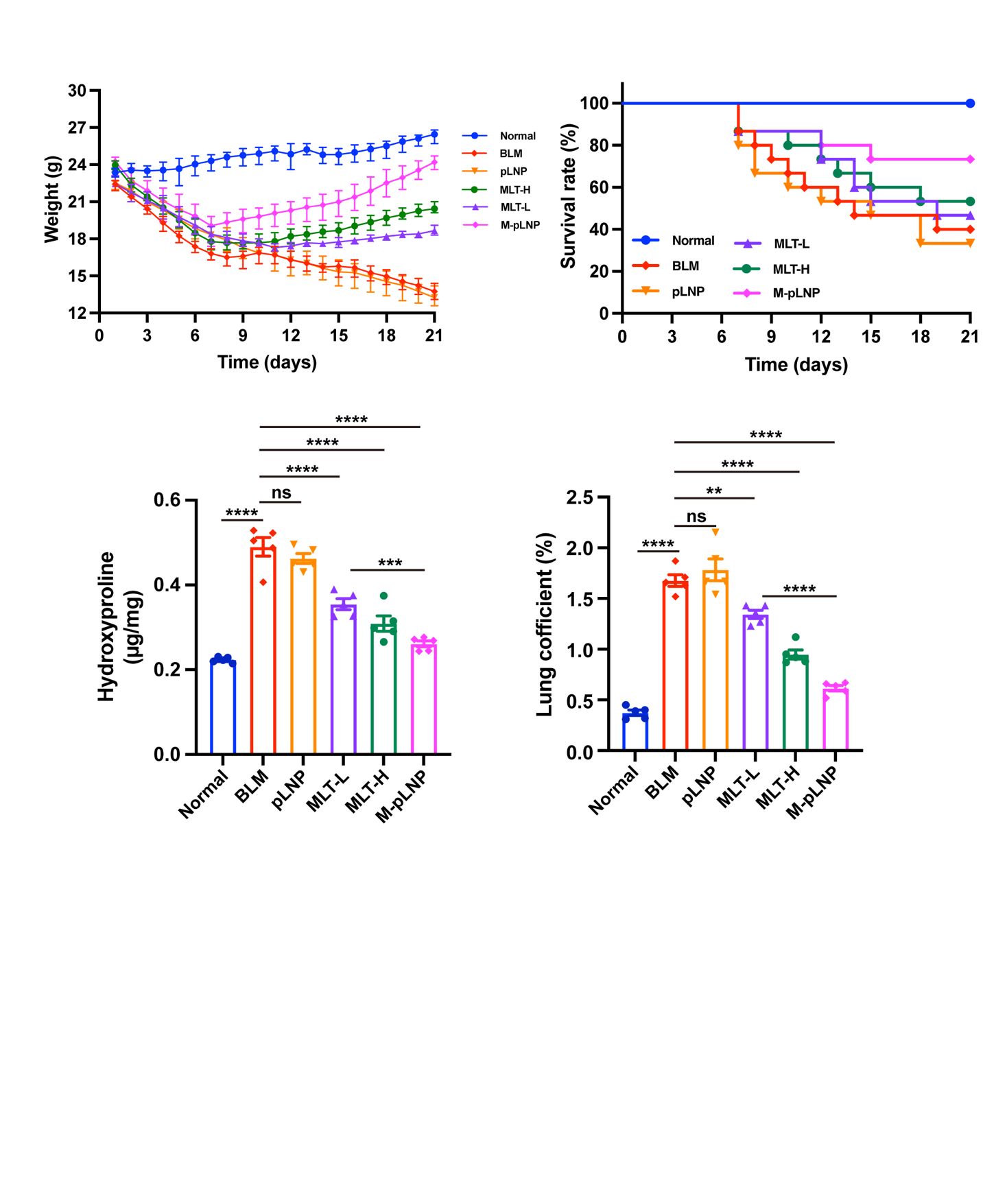


**Fig. S10**. Hydroxyproline levels in lung tissue. Data are presented as the mean ± SEM (n = 5); ns: not significant, ****p* < 0.001, *****p* < 0.0001.
